# Selection and validation of potato candidate genes for maturity corrected resistance to *Phytophthora infestans* based on differential expression combined with SNP association and linkage mapping

**DOI:** 10.3389/fgene.2015.00294

**Published:** 2015-09-23

**Authors:** Meki S. Muktar, Jens Lübeck, Josef Strahwald, Christiane Gebhardt

**Affiliations:** ^1^Department for Plant Breeding and Genetics, Max Planck Institute for Plant Breeding ResearchCologne, Germany; ^2^Saka-Pflanzenzucht GmbH & Co. KGWindeby, Germany

**Keywords:** association mapping, candidate genes, differential expression, late blight, marker assisted selection, potato (*Solanum tuberosum* L.), quantitative resistance

## Abstract

Late blight of potato (*Solanum tuberosum* L.) caused by the oomycete *Phytophthora infestans* (Mont.) de Bary, is one of the most important bottlenecks of potato production worldwide. Cultivars with high levels of durable, race unspecific, quantitative resistance are part of a solution to this problem. However, breeding for quantitative resistance is hampered by the correlation between resistance and late plant maturity, which is an undesirable agricultural attribute. The objectives of our research are (i) the identification of genes that condition quantitative resistance to *P. infestans* not compromised by late plant maturity and (ii) the discovery of diagnostic single nucleotide polymorphism (SNP) markers to be used as molecular tools to increase efficiency and precision of resistance breeding. Twenty two novel candidate genes were selected based on comparative transcript profiling by SuperSAGE (serial analysis of gene expression) in groups of plants with contrasting levels of maturity corrected resistance (MCR). Reproducibility of differential expression was tested by quantitative real time PCR and allele specific pyrosequencing in four new sets of genotype pools with contrasting late blight resistance levels, at three infection time points and in three independent infection experiments. Reproducibility of expression patterns ranged from 28 to 97%. Association mapping in a panel of 184 tetraploid cultivars identified SNPs in five candidate genes that were associated with MCR. These SNPs can be used in marker-assisted resistance breeding. Linkage mapping in two half-sib families (*n* = 111) identified SNPs in three candidate genes that were linked with MCR. The differentially expressed genes that showed association and/or linkage with MCR putatively function in phytosterol synthesis, fatty acid synthesis, asparagine synthesis, chlorophyll synthesis, cell wall modification, and in the response to pathogen elicitors.

## Introduction

The late blight disease caused by the oomycete *Phytophthora infestans* (Mont.) de Bary, is one of the most important bottlenecks of potato (*Solanum tuberosum* L.) and tomato (*Solanum lycopersicum*) cultivation worldwide (Guenthner et al., 2001). Since its first pandemic outbreak in the middle of the nineteenth century, and despite 100 years of resistance breeding, *P. infestans* remains the most destructive pathogen of potato (Yoshida et al., 2013). *P. infestans* causes sporulating lesions on foliage, stems and tubers, which under favorable weather conditions spread within days throughout a field of susceptible cultivars. If not controlled, late blight epidemics can completely destroy the crop within few weeks. Currently, *P. infestans* is controlled by frequent applications of fungicides, which is environmentally unsafe and costly (Guenthner et al., 2001). In addition, continuous fungicide exposure promotes the emergence of fungicide resistant strains of *P. infestans*. Improving the potato's genetic resistance is the option to reduce chemical control and hence, production costs and environmental hazards.

The quest for late blight resistant cultivars began after the Irish Famine in the 1840s. Single, dominant genes conferring complete resistance to specific races of *P. infestans* (*R* genes) were identified in wild potato species and introgressed into advanced cultivars. However, this type of resistance proved not durable, as new *P. infestans* races having virulence alleles compatible with host resistance genes evolved after few years of widespread cultivation of the resistant varieties. The alternative to single *R* genes is polygenic or quantitative resistance to late blight, which is in essence the natural, quantitative variation of a compatible host-pathogen interaction. It allows the pathogen to multiply in certain degree but slows down the rate of disease progression, thereby reducing the selection pressure on the pathogen. This type of resistance is more durable as the pathogen has to undergo multiple mutations to overcome polygenic resistance. Quantitative resistance is also largely race unspecific (Ross, 1986; Wastie, 1991; Darsow, 2014).

Breeding for high field resistance to late blight is challenging and requires multiple-year and location trials. Resistance to late blight has to be combined with other agronomic characters such as high tuber yield, resistance to other pests and pathogens, culinary qualities, and early plant maturity. Unfortunately, high field resistance to late blight is correlated with late plant maturity, which is an undesirable trait (Visker et al., 2003; Bormann et al., 2004; Darsow, 2014). Quantitative resistance to late blight depends on the developmental stage or maturity of the plant, which in turn depends on day length (Kloosterman et al., 2013; Darsow, 2014). The reason for this might be that the same genes which condition late maturity have pleiotropic effects on quantitative resistance, or that the genes controlling late maturity and quantitative resistance are different but physically closely linked and therefore co-inherited (Bormann et al., 2004). However, there is evidence that quantitative resistance to late blight is not entirely explained by the maturity effect (Bormann et al., 2004; Bradshaw et al., 2004; Pajerowska-Mukhtar et al., 2009; Darsow, 2014). The objective of our research is the discovery of genes that condition field resistance to late blight not compromised by late plant maturity. DNA polymorphisms associated with resistance to late blight, which are located either directly in such genes or physically closely linked with them, can be used for the early diagnosis of superior alleles in breeding populations, thereby increasing precision and efficiency of quantitative resistance breeding (Gebhardt, 2013).

The first diagnostic markers for quantitative resistance to late blight resulted from association mapping in populations of tetraploid varieties and breeding clones based on DNA polymorphisms in candidate genes (Gebhardt et al., 2004; Malosetti et al., 2007; Pajerowska-Mukhtar et al., 2009; Odeny et al., 2010). Outstanding with respect to association with maturity corrected resistance (MCR) were two single nucleotide polymorphisms (SNPs) in the *StAOS2* gene, which encodes an allene oxide synthase, a key enzyme in the biosynthesis of the jasmonate plant hormones (Pajerowska-Mukhtar et al., 2008; Kombrink, 2012). The candidate genes considered were genes known or expected to function in pathogen recognition (*R* genes and *R* gene homologs), defense signaling and defense responses (Gebhardt, 2013). However, the molecular basis of quantitative resistance to pathogens is largely unknown and might involve additional genes, pathways and mechanisms that are not discovered when studying *R* gene mediated resistance (Poland et al., 2009). Comparative transcript profiling in combination with next generation sequencing technology (van Dijk et al., 2014) is a novel approach to discover *de novo* candidate genes for quantitative resistance in general and quantitative resistance to *P. infestans* in particular. To this purpose we have compared the transcriptomes of pooled, heterozygous, tetraploid potato genotypes that differed for mean late blight resistance corrected for the maturity effect (MCR) (Draffehn et al., 2013). Transcript levels in the genotype pools before and after controlled infection with *P. infestans* were quantified by SuperSAGE (serial analysis of gene expression) analysis. SuperSAGE generates from each transcript present in a biological sample 26 base pair sequence tags, the frequency of which is proportional to the frequency of the corresponding transcript. Individual tag frequencies are determined by next generation sequencing of complex tag libraries. Transcripts are identified by mapping the tags to annotated EST (expressed sequence tag) collections and/or annotated genome sequences. SuperSAGE permits the simultaneous detection and quantification of the transcriptome both from the host and the pathogen in infected samples (Matsumura et al., 2003). Our SuperSAGE comparison of tag frequencies in potato genotype pools with contrasting MCR levels revealed 806 differential transcripts from approximately 720 genes (Draffehn et al., 2013).

In this follow up study, novel functional candidate genes were selected from 806 differential transcripts identified by SuperSAGE and subjected to validation of their differential expression in different genotype pools and independent infection experiments. The suitability of differentially expressed genes as diagnostic markers for late blight resistance was tested by association mapping, using as markers single nucleotide polymorphisms (SNPs) in the corresponding loci.

## Materials and methods

### Plant materials

For association mapping, the PIN184 population was used, which consisted of 184 tetraploid potato genotypes (Pajerowska-Mukhtar et al., 2009). The PIN184 population has been assembled from breeding materials of the breeding companies Böhm-Nordkartoffel Agrarproduktion OHG (BNA) and SaKa-Pflanzenzucht GmbH & Co. Kg (SKP). The population has been phenotyped for quantitative resistance to complex races of *P. infestans* and for plant maturity in 3 years in the field at Ebstorf (the BNA subpopulation) and Windeby (the SKP subpopulation) as described (Bormann et al., 2004). Field resistance was quantified by the area under disease progress curve (AUDPC) and plant maturity (PM) by scoring from 1 to 9 (1 very late and 9 very early maturing). Maturity corrected resistance (MCR) and adjusted entry means for AUDPC, rAUDPC (relative area under disease progress curve), PM and MCR were calculated from the phenotypic data as described previously (Pajerowska-Mukhtar et al., 2009). Genomic DNA of the PIN184 population maintained at the Max Planck Institute for Plant Breeding Research (Cologne, Germany) was used for SNP genotyping by amplicon sequencing and pyrosequencing.

For linkage analysis, two tetraploid half-sib families SL1 and SL2 comprising 76 and 35 F1 genotypes, respectively (SL genotypes), were used. They originated from the crosses Phy20 × Phy14 (SL1 family) and Phy20 × Phy16 (SL2 family) (Draffehn et al., 2013). The pollen parents Phy14 and Phy16 were quantitative resistant to complex isolates of *P. infestans* whereas the common seed parent Phy20 was susceptible. The SL clones were evaluated in 2010 in the field for AUDPC and PM, and MCR was calculated as described (Pajerowska-Mukhtar et al., 2009). Genomic DNA was extracted from freeze dried leaf samples of each genotype using the DNeasy® Plant mini Kit (Qiagen, Hilden, Germany) according to the manufacturer's instructions.

The 12 most resistant and susceptible genotypes were selected from the 111 SL genotypes based on the MCR values in the field 2010 (Table 1). The mean between the resistant and susceptible group was significantly different at *p* < 0.001 for AUDPC and MCR and not significant for PM (*p* > 0.05). These 24 genotypes were infected under controlled conditions with a complex isolate of *P. infestans* and used for expression analysis of candidate genes.

**Table 1:** **Means and standard deviations of resistant and susceptible groups of SL genotypes for AUDPC, PM, and MCR**.

**Group**	**Mean AUDPC (*SD*)**	**Mean PM (*SD*)**	**Mean MCR (*SD*)**
Resistant (*n* = 12)	38.81 (39.03)	5.08 (1.55)	−93.19 (30.32)
Susceptible (*n* = 12)	219.51 (27.38)	4.50 (1.35)	71.62 (14.59)

### Controlled infection of SL genotypes with *P. infestans*

*In vitro* plantlets of the 24 selected SL genotypes were transferred to the greenhouse, propagated in pots for 5 to 6 weeks and then transferred to a growth chamber (Snijders Scientific B.V., Tilburg, Netherlands) set at 80% humidity, 100% light, 16 h light at 22°C and 8 h dark at 18°C and acclimated for few days before infection with *P. infestans*. Four plants per genotype were grown for four different treatments. One plant was used to collect leaf tissue immediately prior to inoculation (T0), the second plant was sprayed with water as negative control to check that water alone did not cause any blight symptoms. The remaining two plants were inoculated with a complex field isolate of *P. infestans*. The isolate was virulent on plants carrying the major late blight resistance genes *R1, R2, R3, R4, R6, R7, R10*, and *R11*, and avirulent on *R8* and *R9* carrying plants.

*P. infestans* mycelium was weekly propagated on fresh leaflets of the susceptible cultivars Grata and Granola. Sporangia were collected in a sterile beaker by rinsing the infected leaves with sterile tap water. The concentration was adjusted to 20 sporangia/μl and zoospores were released after 1–2 h incubation in the dark at 4°C.

The third, fourth, and fifth compound leaves (counting from the top) of each plant were marked with rings and sprayed with the sporangia/zoospore suspension or water two to three times using a 20 ml spraying bottle (cat. No10007245, neoLab, Heidelberg, Germany). Infected and control plants were covered with transparent plastic bags to maintain high humidity. On the second (T2) and third (T3) day after inoculation, the fourth and fifth leaves were collected using a sterile blade, rapped in aluminum foil, immediately frozen in liquid nitrogen and stored at −80°C until use. Leaf samples were collected in the morning between 9 and 10 a.m. After collecting the leaf samples, the plants were kept in the growth chamber for 1 week to visually monitor *P. infestans* growth and symptom development on the third compound leaf of each genotype. Disease progression was scored as percent infection (Henfling, 1982) for three consecutive days starting at day 3. AUDPC values were calculated according to Fry (1978). In addition, *P. infestans* growth was monitored in the infected leaf samples at T2 and T3 by qRT-PCR (three replicates each) of the *P. infestans* ribosomal gene *RL23a* (PITG_02694) as described (Draffehn et al., 2013). The infection experiments were repeated five times with fresh plants and inoculum. The three most successful experiments were used for expression analysis of candidate genes.

### Construction of RNA pools and cDNA synthesis

Total RNA was extracted from approximately 100 mg non-infected and infected leaf samples using the TRIzol RNA isolation kit (Ambion®) according to the supplier's protocol. Four sets of RNA pools (Pools_1 to Pools_4) (Supplemental File S1) were prepared by combining equal amounts of RNA from 3 to 12 SL genotypes. Each set consisted of six RNA pools in three biological replicates (three infection experiments): a resistant (R) and a susceptible (S) genotype pool, each at the infection time points T0, T2, and T3.

Pools_1 were constructed by pooling RNA from the 12 most resistant and susceptible SL genotypes that were selected based on MCR in the field in 2010 (see above). In Pools_2, RNA was pooled from the six most resistant and susceptible SL genotypes, based on the quantification by qRT-PCR of *P.infestans* growth after controlled infection. The pooling in Pools_3 was based on the AUDPC values of the third compound leaf of each infected plant. RNA was pooled from the five most resistant and susceptible SL genotypes. Pools_4 were assembled from RNA of three resistant and susceptible SL genotypes, based on MCR in the field 2010 plus the genotype of two SNPs in the *StAOS2* gene, which were associated with MCR in the PIN184 population (Pajerowska-Mukhtar et al., 2009). The three quantitative resistant genotypes were homozygous for the haplotype *StAOS2_A*_691_*C*_692_associated with increased resistance, while the three susceptible genotypes had one copy of haplotype *StAOS2_A*_691_*C*_692_ and three copies of the haplotype *StAOS2_G*_691_*G*_692_ associated with increased susceptibility. The concentration of the pooled RNA was measured using the Nanodrop ND-1000 spectrophotometer (Peclab, Erlangen) before performing cDNA synthesis. First strand cDNA was synthesized using the Maxima H Minus First Strand cDNA Synthesis Kit (Thermo Scientific) following the supplier's protocol.

### Selection of candidate genes

The genes analyzed in this study were selected based on differential expression detected by SuperSAGE (serial analysis of gene expression) analysis, which generates 26 bp tags from the transcripts present in a biological sample (Matsumura et al., 2010). The transcript level is proportional to the tag counts or, when more than one tag matches to the same transcript, by the hit counts, that means the sum of all tags matching to the same transcript. Normalized hit counts (hpm = hits per million) have been compared between three genotype pools A1, A2, and B2 with different levels of MCR. The genotype pools were generated by combining 6 to 14 SL genotypes originated from the same crosses Phy20 × Phy14 and Phy20 × Phy16 (Draffehn et al., 2013) as used in this study. Eight of the 24 SL genotypes used to construct the RNA pools were also included in the genotype pools A1, A2, and B2. The mean MCR of the genotype pool A2 was significantly lower compared to both genotype pools A1 and B2. For simplicity we refer subsequently to genotype pool A2 as the resistant pool and to A1 and B2 as the susceptible pools. Hpm in genotype pool A2 were compared with hpm in pools A1 and B2 before (T0), one (T1) and two (T2) days after controlled infection with *P. infestans*. The details of the genotype pools, infection experiments, SuperSAGE libraries, and data analysis are described by Draffehn et al. (2013). The six comparisons between genotype pools (A2 vs. A1 and A2 vs. B2 at T0, T1, and T2) resulted in approximately 720 genes that were differentially expressed before and after infection with *P. infestans* between genotype pools with different MCR levels (Draffehn et al., 2013). From this set of genes the candidate genes analyzed in this study were selected. Unique 26 bp tags differentially expressed (*p* < 10^−4^) in at least five of the six comparisons between groups A2, A1, and B2 were selected. Transcript sequences matching the tags were retrieved from the DFCI (Dana-Farber Cancer Institute) potato EST (expressed sequence tag) database and checked for alignment with the tag sequence. Tags and transcript sequences were matched by BLAST searches to the potato genome reference sequence (version 4.03) at http://solanaceae.plantbiology.msu.edu/. From the matches in the potato genome sequence, the annotated genes aligning with the corresponding tag sequence (minimum 25 of 26 bp) were selected. Genomic DNA sequence, locus name, intron-exon structure and genomic position of the candidate gene were collected from the genome browser. When no match of the tag and transcript sequence was found in the potato genome reference sequence, the transcript sequence from DFCI was used in further analysis. A heatmap was constructed for the differential expression of the selected unique 26 bp tags using a custom made R script.

### Quantitative real time PCR (qRT-PCR)

qRT-PCR was performed on a Mastercycler^ep^ realplex (Eppendorf, Hamburg) or on a CFX384 Touch Real-Time PCR (Bio-Rad) using 2.5 μl of 1:5 diluted (with high purity nuclease free water, Ambion) first strand cDNA and the Power SYBR® Green PCR Master Mix (Applied Biosytems) according to the supplier's protocol. PCR cycling conditions were as described by Draffehn et al. (2013) with annealing temperatures as specified in Supplemental File S2. For standard curve analysis, a serial dilution of 1:10, 1:100, and 1:1000 of the cDNA was prepared. Expression levels were normalized against transcripts of the SAND gene (Czechowski et al., 2005; Expósito-Rodríguez et al., 2008; Draffehn et al., 2013) and averaged across two technical replicates. For quantification of the *P. infestans* transcript RL23a, 2.5 μL of the undiluted first strand cDNA were used. The *P. infestans* RL23a transcript levels were not normalized.

### Pyrosequencing

Pyrosequencing (Ronaghi et al., 1996) was performed using pyromark gold Q96 reagents kit (Qiagen) and a PSQTM 96 MA pyrosequencing instrument (Biotage AB) according to manufacturer's protocols. Gene specific cDNA or genomic DNA fragments between 100 and 300 base pairs containing the SNP of interest were amplified by PCR using the primers listed in Supplemental File S2. The third sequencing primer was used to sequence a short region around the SNP of interest (Supplemental File S2). The PCR reaction was carried out in 25 μl total volume containing 2.5 μl 10x buffer (100 mM Tris-HCL pH 8.3, 500 mM KCl, 15 mM MgCl_2_, 1% Triton X-100), 0.2 mM dNTP Mix, 0.4 μM each forward and reverse primers, 0.5 U Taq DNA Polymerase (Ampliqon), high purity water (Merck KGaA, Darmstadt, Germany) and 50 ng template DNA. The PCR reaction was run with 2 min initial denaturation at 94°C, followed by 50 cycles with 45 s denaturation at 94°C, 45 s annealing at the temperature specified for each primer pair (Supplemental File S2), and extension for 1 min at 72°C. At the end of the cycles, the reaction was run for 10 min at 72°C. Reactions were generally performed in a Labcycler (SensoQuest GmbH, Göttingen).

### Amplicon sequencing and SNP calling

Gene specific primers were designed (Supplemental File S2) using primer-BLAST in NCBI at http://www.ncbi.nlm.nih.gov/tools/primer-blast/. Primers were preferentially located in exons and in few cases in introns. DNA fragments between 400 and 1300 bp were amplified. The standard PCR reaction was carried out in 25 μl total volume containing 2.5 μl 10x buffer (100 mM Tris-HCL pH 8.3, 500 mM KCl, 1% Triton X-100), 0.2 mM dNTP Mix, 2.5 mM MgCl_2_, 0.2 μM each forward and reverse primers, 0.2 U Taq DNA Polymerase (Ampliqon), high purity water (Merck KGaA, Darmstadt, Germany), and 50 ng template DNA. The PCR reaction was run with 2 min initial denaturation at 94°C, followed by 35 to 40 cycles with 40 s denaturation at 94°C, 45 s annealing at the temperature specified in Supplemental File S2, and extension for 1:30 min at 72°C. At the end of the cycles, the reaction was run for 10 min at 72°C. Amplicons were custom sequenced at the Max-Planck-Genome-Center Cologne using the dideoxy chain-termination sequencing method, an ABI PRISM Dye Terminator Cycle Sequencing Ready Reaction Kit and an ABI PRISM 3730 automated DNA Sequencer (Applied Biosystems, Weiterstadt, Germany). SNPs were detected by sequence alignment using DNASTAR® Lasergene software Seqman. SNPs were called in dosage dependent manner in the sequence trace files either as AAAA (nulliplex), AAAB (simplex), AABB (duplex), ABBB (triplex), or BBBB (quadruplex) using DAx software (Van Mierlo Software Consultancy, Eindhoven, The Netherlands). The heterozygous groups AAAB, AABB, and ABBB were distinguished based on the relative peak height of each nucleotide in the sequence trace file.

### Population structure and kinship analysis

Population structure and relative kinship in the PIN184 population were estimated based on the genotype of 241 SolCAP SNPs (Hamilton et al., 2011) uniformly distributed on the 12 potato chromosomes (Supplemental File S3). The SNP markers were selected from genome wide genotyping the PIN184 population with the 8.3 k SolCAP genotyping chip (manuscript in preparation). The selected SNPs had a minor allele frequency (MAF) ≥ 10% and no missing data. A Bayesian clustering approach implemented in the STRUCTURE software (Pritchard et al., 2000) was used to assess population structure in the PIN184 population. Burn-in time as well as iteration number were set to 100, 000 with 10 repetitions, testing the probability of 20 subpopulations. An admixture model with correlated allele frequencies was used. The results of the run were uploaded to the software “Structure Harvester” (Earl and vonHoldt, 2012), at http://taylor0.biology.ucla.edu/structureHarvester/ and the most likely number of subpopulations was determined by the log likelihood (Rosenberg et al., 2001) combined with the Evanno method (Evanno et al., 2005). Relative kinship between pairs of genotypes was analyzed using SPAGeDi software (Hardy and Vekemans, 2002) according to Ritland (1996). Negative kinship values between genotypes were automatically set to zero.

### Association analysis

Marker-trait associations were analyzed using TASSEL2.1 software (Bradbury et al., 2007) using a mixed linear model (MLM) which takes into account population structure (Q) and kinship (K) to avoid spurious associations. A *P*-value was generated by fitting each SNP marker into the MLM that has the form;

$$\begin{array}{l} \text{y}=\text{Xb}+\text{Qv}+\text{u}+\text{e}, \end{array}$$

where, y is the vector of the phenotypic values, X is the vector of SNP marker genotypes, b is the vector of marker fixed effects to be estimated, Q is population structure (derived from STRUCTURE analysis), v is a vector of fixed effects due to population structure, u is the vector of random effects due to the K matrix and e is the vector of residuals. A *P* < 0.01 was adopted as threshold for significance.

### Linkage disequilibrium analysis

Pair wise linkage disequilibrium (LD) was analyzed by testing for independence of two loci with a chi-square test using the statistical software R (www.r-project.org). The *P*-values were corrected for multiple testing using the R package “*q*-value,” using the method described by Storey (2003).

### Linkage mapping

Marker-trait linkages were analyzed by analysis of variance (ANOVA) using the linear model function *lm ()*, in R statistical software with the model;

$$\begin{array}{l} \text{Phenotype}=\text{SNP marker}+\text{error} \end{array}$$

with SNP markers in dosage form. A *P* < 0.01 was adopted as threshold for significance.

## Results

In a previous study (Draffehn et al., 2013), we quantified and compared the transcriptomes of pooled, heterozygous, tetraploid potato genotypes selected for contrasting MCR using SuperSAGE (serial analysis of gene expression) technology and next generation sequencing. Multiple comparisons of tag frequencies between the genotype pools with contrasting MCR levels had revealed 806 differential transcripts from approximately 720 genes. This set of potato genes was the basis for selecting novel functional candidate genes for further characterization as described below, with emphasis on reproducibility of differential expression and suitability as diagnostic markers.

### Selection of candidate genes for quantitative resistance against *P. infestans*

**Figure 1:**
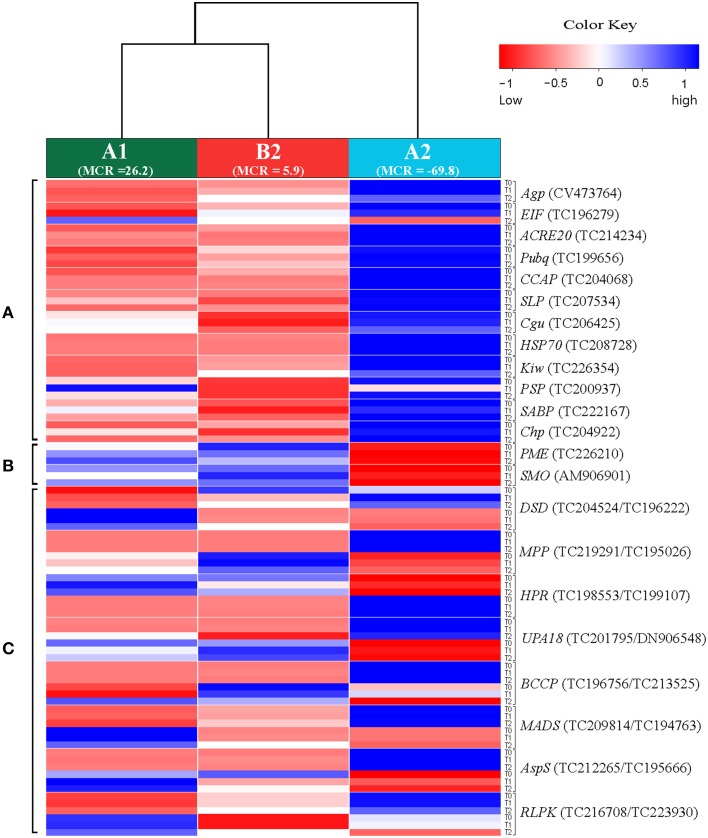
**Heatmap showing the expression of 30 unique SuperSAGE tags representing 22 candidate genes (**Table 2**) in genotype pools A1, B2, and A2**. Columns represent the genotype pools A1, B2, and A2 with mean MCR of each pool shown in parenthesis. Rows represent tags per million (tpm) before (T0), one (T1), and two (T2) days after infection. Gene acronyms (see Table 2) are shown to the right with transcript number in parenthesis. The 12 genes at the top **(A)** were expressed at higher level in the resistant genotype pool A2 compared to both susceptible pools A1 and B2. The two genes in the middle **(B)** were expressed at higher level in pools A1 and B2 compared to A2. The eight genes at the bottom **(C)** showed contrasting expression of pairs of allelic tags. Increasing and decreasing expression is indicated by blue and red color, respectively. The numerical data are shown in Supplemental File S4.

Twenty two of approximately 720 candidate genes were selected based on their differential expression in SuperSAGE between the resistant genotype pool A2 and the susceptible genotype pools B2 and A1 in at least five of six comparisons (A2 vs. A1, and A2 vs. B2 before (T0), one (T1), and two (T2) days after infection, Draffehn et al., 2013) (Figure 1, Supplemental File S4).

Twelve genes were expressed at higher level in the resistant genotype pool A2 compared with both susceptible pools A1 and B2. Vice versa, two genes were expressed at higher level in pools A1 and B2 compared with A2. Six genes were transiently up regulated 1 day after infection (T1) with *P. infestans* (*EIF, ACRE20, SLP, HSP70, SABP, Chp*), one (*PME*) was consistently up and three (*Agp, PSP, SMO*) were down regulated. The remaining four genes (*Pubq, CCAP, Cgu, Kiw*) showed minor or inconsistent responses to infection. For each of the 14 genes, a unique 26 bp tag (Table 2) showed differential tag counts in five or all six comparisons (Supplemental File S4). The expression of these 14 genes was evaluated by qRT-PCR in four sets of genotype pools and three independent infection experiments (see below).

**Table 2:** **Summary of 22 selected candidate genes: annotation, acronym, representative tag sequence, transcript number, chromosomal position, and locus identification**.

**Annotation**	**Acronym**	**The 26 bp tag sequence**	**Transcript number**	**Chr**.	**Locus**
Arabinogalactan protein	*Agp*	GATCATTGAGATGAGGAGTTTGACGT	CV473764	10	PGSC0003DMG400008381
Eukaryotic translation initiation factor 4e type	*EIF*	CATGTAATATGAGCTAACAATTAGAT	TC196279	10	PGSC0003DMG400023664
Avr9/Cf-9 rapidly elicited protein 20	*ACRE20^a^*	GATCTTAAGGGTATGGTTAAAGAAGG	TC214234	10	PGSC0003DMG400025023
Polyubiquitin	*Pubq^a^*	CATGATGGCTGTGCGCTTTGTTGTTT	TC199656	NA	no match
Clathrin coat assembly protein AP17	*CCAP^a^*	CATGTTGATGTGGCCTCATTTAATAC	TC204068	4	PGSC0003DMG400009862
Subtilisin-like protease	*SLP*	CATGAGAGCTATGTTTTTAATTATGG	TC207534	1	PGSC0003DMG400006781
Conserved gene of unknown function	*Cgu*	CATGAACAGAGTGTGTATTTGTATAG	TC206425	12	PGSC0003DMG400004981
Heat shock cognate 70 kDa protein	*HSP70*	CATGTTTTTGGTTTCGTCAGTTAGTT	TC208728	9	PGSC0003DMG400008917
Kiwellin	*Kiw*	GATCCAGTGAGCAGTTCAGAGTCAGT	TC226354	11	PGSC0003DMG400008099
				NA	PGSC0003DMG400043121
				2	PGSC0003DMG400021473
Photosystem II core complex proteins	*PSP*	GATCATCATAGTTTGTAATATTTGGA	TC200937	10	PGSC0003DMG400007201
Salicylic acid-binding protein 2	*SABP*	GATCCATCTCTACATCTTCTACTCTT	TC222167	2	PGSC0003DMG400013101
Chloroplast protease	*Chp^a^*	GATCTAGAGTATAAACAATAGACTAT	TC204922	7	PGSC0003DMG400017311
Pectin methyl esterase	*PME^a^*	CATGTTAATATTAACTATTGTGTTTA	TC226210	3	PGSC0003DMG400009178
Squalene monooxygenase	*SMO^a^*	CATGATTGATTACACTATTAACTGGA	AM906901	4	PGSC0003DMG400004923
Delta (7)-sterol-C5 (6)-desaturase	*DSD^a^*	CATGATTTGCA[C/A]TCAAGGATGTTCCT	TC204524 /TC196222	2	PGSC0003DMG400026401
Magnesium-protoporphyrin IX monomethyl ester [oxidative] cyclase, chloroplast	*MPP^a^*	CATGTTCATTGTTGTAAGTT[G/A]TATAG	TC219291/TC195026	10	PGSC0003DMG400007188
Hydroxypyruvate reductase	*HPR^a^*	TCC[A/G]TCCTAATTATAGCAAAATCATG	TC198553/TC199107	1	PGSC0003DMG400006186
Up-regulated by AvrBs3	*UPA18^a^*	GATCAGAAGCGTAGTGGGAAAAT[A/G]GG	TC201795 /DN906548	9	PGSC0003DMG400006079
Biotin carboxylase carrier protein	*BCCP^a^*	GATCGGTCAGGAACCAT[T/C]GTTGAGGT	TC196756/TC213525	5	PGSC0003DMG401023454
MADS-box transcription factor 16	*MADS*	GATCAAGATGA[T/C]TCCTCAAATGCATC	TC209814/TC194763	4	PGSC0003DMG400009363
Asparagine synthetase	*AspS^a^*	GATCAAAACCATATA[T/G]AGGTTTTGAA	TC212265/TC195666	6	PGSC0003DMG400004170
Receptor-like protein kinase	*RLPK^a^*	CATGAAACTAAGA[T/G]A[G/A]TATTTCTTGT	TC216708 /TC223930	3	PGSC0003DMG400015157

*The alternative nucleotides of allelic tags are shown in brackets*.

a *Gene was selected for association mapping. NA = chromosome position is not known*.

Eight further genes were represented by two SuperSAGE tags each that differed by one or two nucleotides, matched to the same locus and therefore represented allelic variants of the eight genes (Table 2). The allelic tags showed contrasting expression between resistant and susceptible pools. One tag was expressed at higher level in the resistant pool A2 while the other was expressed at higher level in susceptible pools A1 and B2 (Figure 1, Supplemental File S4). Both allelic tags of two (*MPP, HPR*) and one gene (*AspS*) were down and up regulated, respectively, upon infection with *P. infestans*. The response to infection of the allelic tags of the remaining five genes was inconsistent. The tag SNPs in *MPP* and *RLPK* were non-coding, as they were located in the 3' untranslated region of the gene. The tag SNPs caused synonymous changes in *DSD, HPR, BCCP* and *AspS*, and non-synonymous changes in *UPA18* and *MADS*, resulting in a non-conservative (methionine/isoleucine) and a conservative (aspartate/glutamate), respectively, amino acid change (Supplemental Files S5A,B). Expression and allele frequency of the allelic tags was analyzed by pyrosequencing.

**Figure 2:**
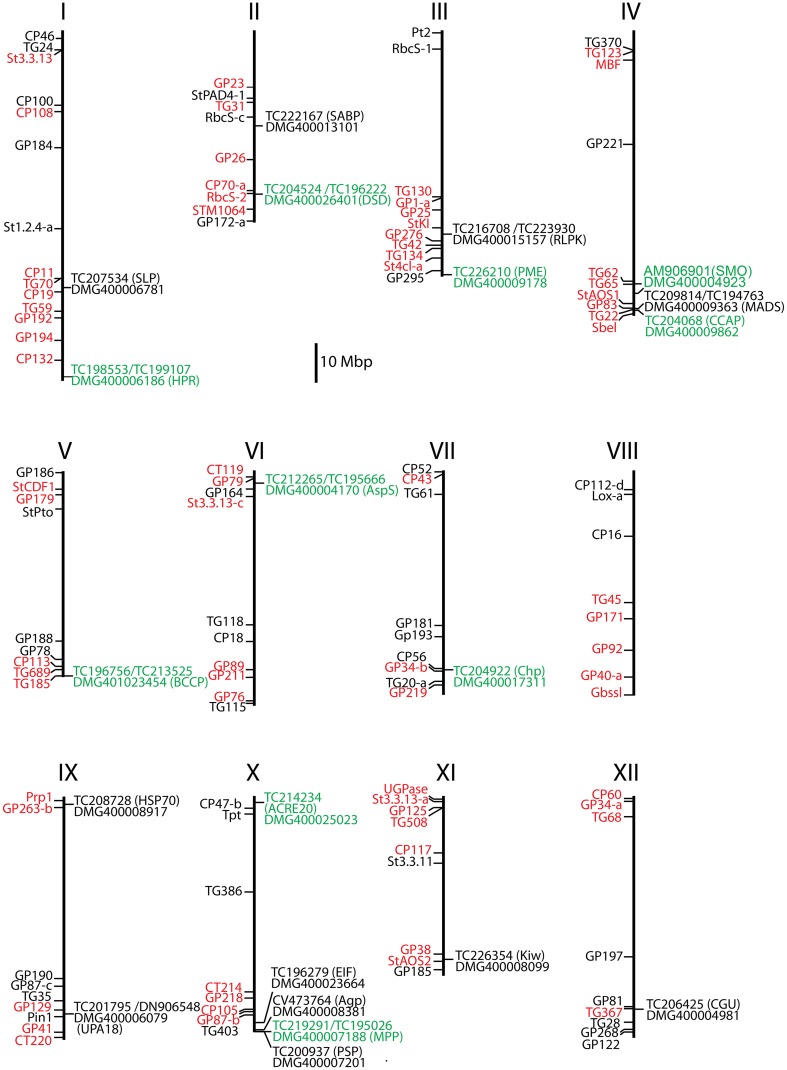
**Positions of 21 selected candidate genes on the physical maps of the 12 potato chromosomes**. Gene *Pubq* was not present on the physical map. RFLP (restriction fragment length polymorphism) markers linked to resistance QTLs are shown in red and additional anchor markers in black to the left of each chromosome map (Gebhardt, 2013). Candidate locus name, matching transcript number, and gene acronyms (in parenthesis) are shown to the right. Loci where one or more SNPs were associated or linked with MCR and rAUDPC are highlighted green.

With two exceptions (*Pubq, Kiw*) the loci corresponding to the differential tags were unambiguously identified by *in silico* mapping of tag and transcript sequences to the potato genome sequence version 4.03 (Sharma et al., 2013) (Table 2, Figure 2). No match was found for *Pubq* in the genome sequence. The tag matching to the locus PGSC0003DMG400008099 (Kiwellin) mapped equally well to two additional loci, PGSC0003DMG400021473, a short gene fragment annotated as “late blight resistance protein” and PGSC0003DMG400043121, a “Hypothetical gene of unknown function” with unknown genomic position. The locus PGSC0003DMG400008099 (Kiwellin) was chosen for further analysis. It is a member of a clustered family of six “Kiwellin” genes (*KiTH-2*) on chromosome XI, one of which (PGSC0003DMG400008101) was strongly induced upon infection with *P. infestans* and consistently expressed at higher level in resistant genotype pool A2 vs. both susceptible pools A1 and B2 (Draffehn et al., 2013).

### Reproducibility of differential expression

**Figure 3:**
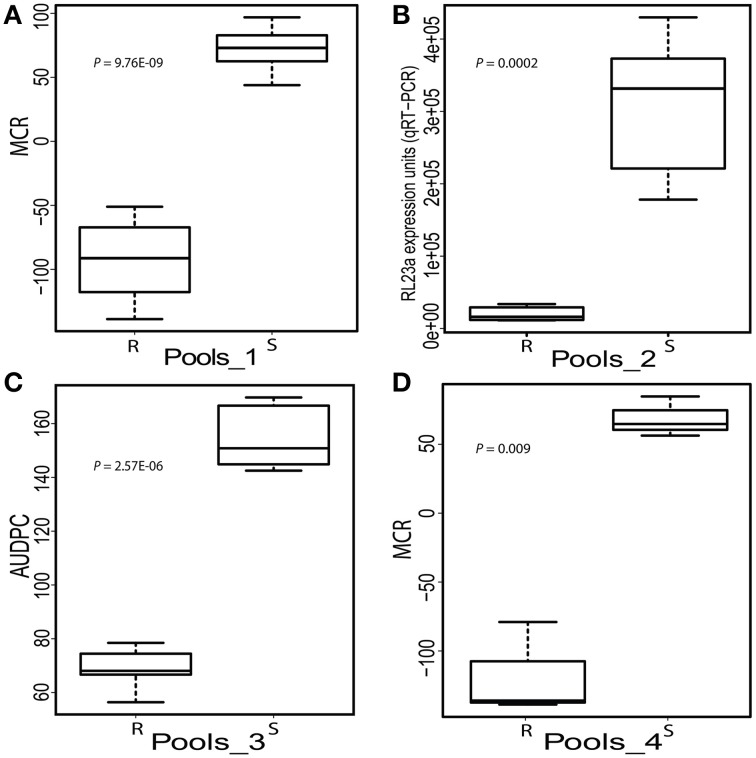
**Boxplots showing the phenotypic means of the SL genotypes selected for high (R) and low (S) resistance to *P. infestans* assessed by four different methods. (A)** Maturity corrected resistance (MCR, evaluated in the field 2010) of Pools_1 (*n* = 12). **(B)** Expression units of the *P. infestans* ribosomal gene *RL23a* (2 and 3 days after controlled infection) in Pools_2 (*n* = 6). **(C)** Area under disease progress curve (AUDPC evaluated between day 3 and 5 after controlled infection) of Pools_3 (*n* = 5). **(D)** MCR as in A of pools_4 (*n* = 3). The individuals in Pools_4 differed for the genotype of SNPs 691 and 692 at the *StAOS2* locus (Pajerowska-Mukhtar et al., 2009).

Expression of the 22 candidate genes in response to controlled infection with *P. infestans* was analyzed in four sets of RNA pools that were assembled from 24 SL genotypes with contrasting quantitative resistance phenotypes (Figure 3, Supplemental File S1). Pools_1 differed for MCR evaluated in the field (Figure 3A), Pools_2 for growth of *P. infestans* measured by qRT-PCR of the *RL23a* ribosomal gene 2 and 3 days after controlled infection (Figure 3B) and Pools_3 for AUDPC evaluated visually from the third to the fifth day after controlled infection (Figure 3C). Pools_4 had contrasting MCR levels as Pools_1 and in addition contrasting genotypes at the *StAOS2* locus (Figure 3D).

**Figure 4:**
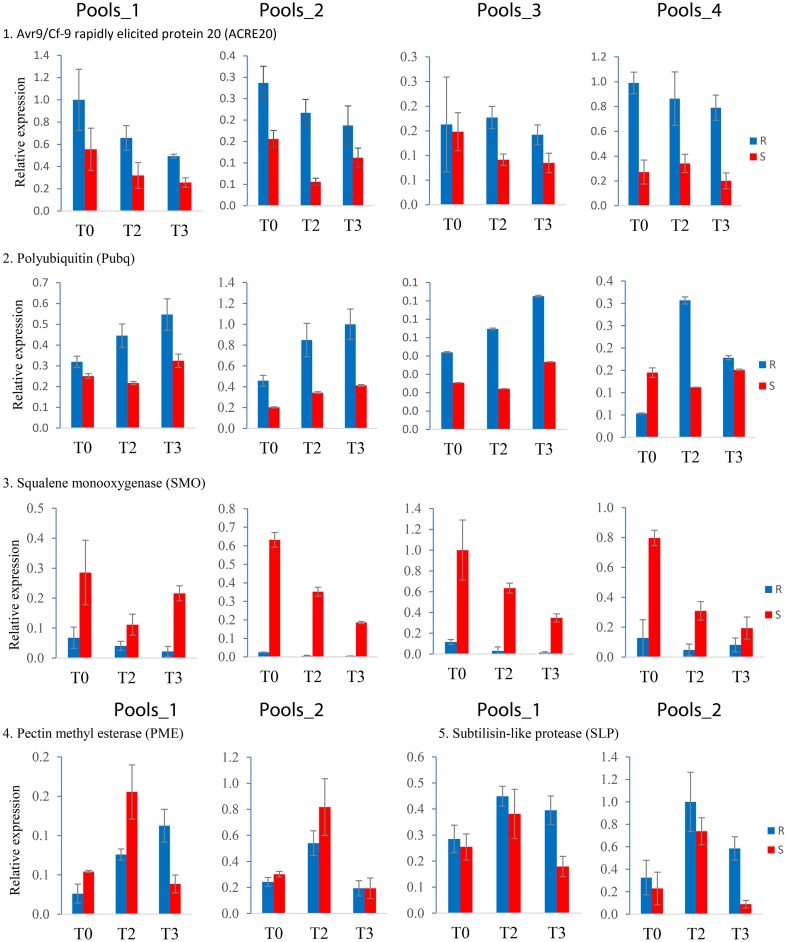
**Expression patterns obtained by qRT-PCR of the five most reproducible candidate genes**. Resistant (R) and susceptible (S) pools are shown as blue and red bars, respectively, at infection time points T0, T2, and T3. Expression of *ACRE20, Pubq*, and *SMO* was quantified in Pools_1, _2, _3, and _4, of *PME* and *SLP* in Pools_1 and _2. The bars represent the data from one representative out of three independent infection experiments. Error bars were derived from two technical replicates.

Expression of 14 genes was analyzed by qRT-PCR in three independent infection experiments (biological replicates). Six genes were tested in all four pools and eight were tested in Pools_1 and Pools_2. Measuring expression in all four sets of pools yielded 72 expression values: four each resistant (R) and susceptible (S) pools at T0, T2, T3, and three biological replicates. When testing two pools, 36 samples were analyzed. The reproducibility of the differential expression observed in SuperSAGE based on 36 or 18 qualitative comparisons between R and S genotype pools is shown in Table 3. Most consistent with the SuperSAGE results (>70% reproducibility) were the expression levels of *ACRE20, Pubq, SLP, PME*, and *SMO* (representative examples shown in Figure 4). Moderate reproducibility (50–70%) was observed for *Agp, CCAP, Cgu, HSP70, Kiw, PSP*, and *Chp* and low reproducibility (< 50%) for *EIF* and *SABP*. Contradictory to their expression in SuperSAGE, these genes were expressed at higher level in S than in R pools.

**Table 3:** **Reproducibility of differential expression of 14 candidate genes by qRT-PCR**.

**Gene**	**No. of comparisons between R and S genotype pools**	**No. of comparisons consistent with SuperSAGE**	**Percent reproducibility**
*Agp*	36	18	50
*EIF*	36	10	28
*ACRE20*	36	32	89
*Pubq*	36	34	94
*CCAP*	36	22	61
*SLP*	18	13	72
*Cgu*	18	9	50
*HSP70*	18	12	67
*Kiw*	18	10	56
*PSP*	18	10	56
*SABP*	18	8	44
*Chp*	18	9	50
*PME*	18	13	72
*SMO*	36	35	97

Twelve genes responded to infection with *P. infestans* similar as in SuperSAGE, taking into account that absolute expression levels and infection kinetics can vary considerably between independent infection experiments. Contradictory results were obtained for *ACRE20* and *Cgu* transcripts. *ACRE20* was transiently up regulated upon infection in SuperSAGE but consistently down regulated in the samples analyzed in this study. Vice versa, *Cgu* was up regulated at T1 and down regulated at T2 in SuperSAGE, while it was continuously up regulated in this study (Supplemental File S4).

**Figure 5:**
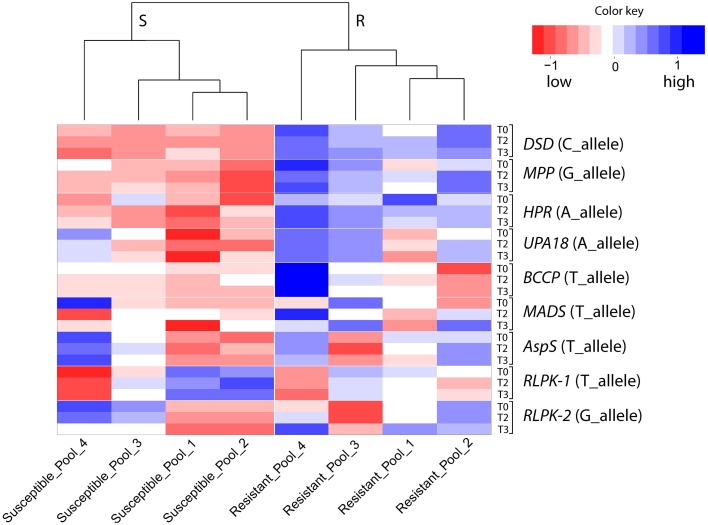
**Heatmap showing SNP allele specific expression in resistant and susceptible pools at infection time points T0, T2, and T3 as quantified by pyrosequencing**. Columns represent the resistant (R) and susceptible (S) genotype pools. Rows represent the expression (average of three infection experiments) at T0, T2, and T3 of the SNP allele that was expressed at higher level in the resistant pool A2 compared with pools A1 and B2 in SuperSAGE (Supplemental File S4). The eight genes with contrasting allele expression are indicated to the right. Increasing and decreasing expression is indicated by blue and red colors, respectively. Numerical data are shown in Supplemental File S6.

The relative expression of alternative SNP alleles in 16 SuperSAGE tags matching to eight candidate genes was quantified by pyrosequencing, using cDNA templates from 72 pooled RNA samples (R and S pools of Pools_1, _2, _3, and_4, three time points, three infection experiments) (Supplemental File S6). The expression in R and S pools of the “resistant” allele (the allele expressed at higher level in the resistant pool A2 compared with susceptible pools A1 and B2 in SuperSAGE) is shown by a heatmap (Figure 5). Resistant and susceptible genotype pools were clearly separated. Pools_1 and _2 were most closely related, whilst Pools_4 were the most distinct.

**Figure 6:**
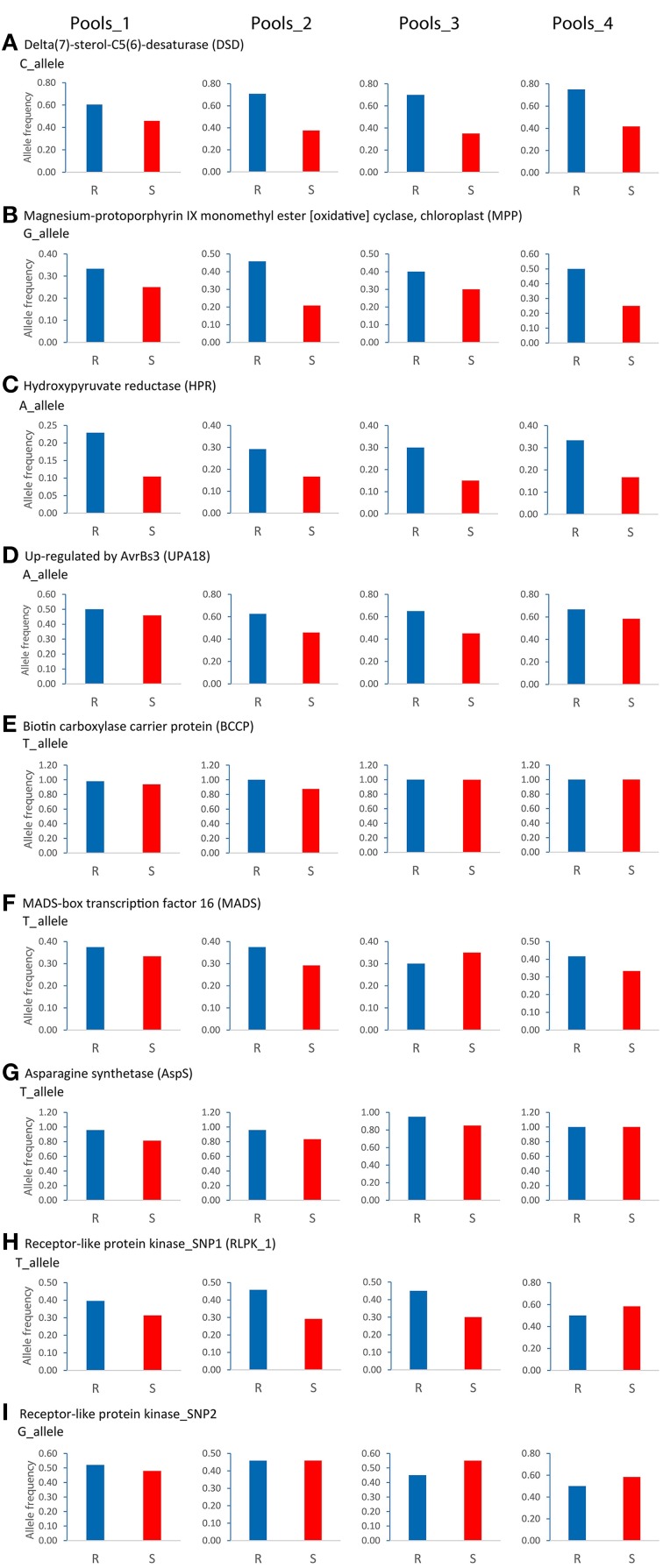
**Genomic allele frequency in resistant (R) and susceptible (S) genotype pools of nine (A–I) SNP allele-pairs with contrasting expression in SuperSAGE**. The relative allele frequency was determined by pyrosequencing on genomic DNA of the 24 SL genotypes used for constructing Pools_1, _2, _3, and _4. The relative frequency of the SNP allele that was expressed at higher level in the resistant pool A2 compared with pools A1 and B2 in SuperSAGE (Supplemental File S4) is shown as blue and red bar in R and S genotype pools, respectively.

Reproducibility of the SuperSAGE expression pattern was assessed by 36 qualitative comparisons between R and S pools (Table 4). The results for the genes *DSD, MPP, HPR*, and *UPA18* were highly consistent with the differential expression in SuperSAGE in all four sets of genotype pools (>80% reproducibility): the “resistant” allele was expressed at higher level in R pools compared with S pools (Figure 5, Supplemental File S6). In these four cases the genomic allele frequency of the “resistant” allele was also higher in R compared to S pools (Figure 6), indicating that the observed differential expression was in part or on the whole due to the differential allele frequency in R and S pools. Results were less consistent for genes *BCCP, MADS, AspS*, and *RLPK* (39–64% reproducibility). In the case of *AspS* and *BCCP*, the “susceptible” allele was either absent in some genotype pools (BCCP: Pools_3 and _4; AspS: Pools_4) or its frequency was very low (BCCP: Pools_1 and _2; AspS: Pools_1, _2, and _3). Nevertheless, the expression pattern of *AspS* was completely reproducible in Pools_1 and _2 in accordance with the allele frequency, but not in Pools_3 and _4. Expression of *BCCP* was completely consistent with SuperSAGE only in Pools_4, although only the “resistant” allele was present in both R and S pools. In four cases (Pools_3: *MADS* and *RLPK-2*, Pools_4: *RLPK-1*and *RLPK-2*) the frequency of the “resistant” allele was higher in the S pool than in the R pool (Figure 6).

**Table 4:** **Reproducibility of differential expression by pyrosequencing**.

**Gene**	**No. of comparisons between R and S genotype pools**	**No. of comparisons consistent with SuperSAGE**	**Percent reproducibility**
*DSD*	36	35	97
*MPP*	36	35	97
*HPR*	36	31	86
*UPA18*	36	34	94
*BCCP*	36	22	61
*MADS*	36	23	64
*AspS*	36	20	56
*RLPK-1*	36	14	39
*RLPK-2*	36	18	50

### Association analysis

SNPs in 13 genes were tested for association with MCR, rAUDPC, and PM in the PIN184 population. Ten genes (*ACRE20, Pubq, SMO, DSD, PME, MPP, HPR, UPA18, BCCP, CCAP*) had shown >60% reproducibility of differential expression in R vs. S pools. The expression patterns of the three remaining genes (*AspS, Chp, RLPK*) as determined by qRT-PCR or pyrosequencing were less consistent with the SuperSAGE results. Gene specific primers were developed for amplicon sequencing and SNP detection (Supplemental Files S2, S7). In addition the nine SNPs from eight allelic SuperSAGE tags were genotyped by pyrosequencing in the PIN184 population. None of the latter showed association with rAUDPC, PM or MCR at *p* < 0.01.

Genes *UPA18* and *Pubq* could not be analyzed by amplicon sequencing in the PIN184 population, *Pubq* because it was not polymorphic and *UPA18* because no readable amplicon sequences were obtained due to insertion-deletion polymorphisms. In the amplicons derived from the remaining 11 genes, the tetraploid genotypes of 129 bi-allelic and 2 tri-allelic SNPs were called. Amplicon sequences, SNP positions and nucleotide alleles are shown in Supplemental File S7.

**Figure 7:**
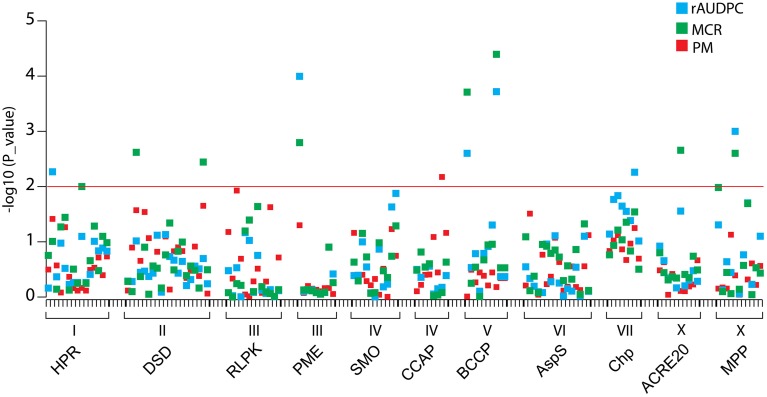
**Association plot of SNPs at 11 candidate loci**. SNPs are plotted on the x-axis and the –log_10_ of the *p*-values on the y-axis. The traits rAUDPC, MCR, and PM are shown by blue, green and red squares, respectively. The threshold *p*-value *p* = 0.01 is indicated by the horizontal line. Gene acronyms and chromosome positions are shown below the X-axis.

Of 131 SNP markers called in the amplicon sequences, nine SNPs at seven loci were associated at *p* < 0.01 with MCR or rAUDPC or both (Figure 7, Table 5). The total variance explained by a single SNP ranged from 6 to 13 %. The strongest association with MCR was observed for BCCP_SNP1381, explaining 13% of the variation. Only one SNP in the *CCAP* locus was associated with PM. The two *DSD* SNPs and the two *BCCP* SNPs were in strong LD with each other, forming haplotypes. Only the two associated *BCCP* SNPs and MPP_SNP2580 showed weak LD with the SNP distinguishing the allelic tags (Supplemental File S8). The two associated *BCCP* SNPs caused both the non-conservative amino acid change serine to proline (Supplemental File S5C).

**Table 5:** **SNPs associated with MCR, rAUDPC, and/or PM in the PIN184 population, allele frequencies, and allele effects**.

**Locus**	**Chr.^a^**	**Marker**	**Alleles**	**Allele frequency [%]**	**MCR *P*-value (*R*^2^)**	**rAUDPC *P*-value (*R*^2^)**	**PM *P*-value (*R*^2^)**
*Hydroxypyruvate reductase (HPR)*	I	HPR_SNP4438	*T/C*	71/29	NS^b^	0.0054 (0.08)↓/↑	NS
*Delta(7)-sterol-C5(6)-desaturase (DSD)*	II	DSD_SNP1516	*T/G*	64/36	0.0024 (0.09)↓/↑^c^	NS	NS
		DSD_SNP3168	*A/T*	75/25	0.0036 (0.06)↓/↑	NS	NS
*Pectin methyl esterase (PME)*	III	PME_SNP4496	*C/A*	84/16	0.0016 (0.07)↓/↑	1.01E-04 (0.10)↓/↑	NS
*Clathrin coat assembly protein AP17 (CCAP)*	IV	CCAP_SNP9879	*A/C*	96/6	NS	NS	0.0067 (0.05)↓/↑
*Biotin carboxylase carrier protein (BCCP)*	V	BCCP_SNP1121	*T/C*	88/12	1.95E-04 (0.11)↓/↑	0.0025 (0.09)↓/↑	NS
		BCCP_SNP1381	*T/C*	78/22	4.78E-05 (0.13)↓/↑	1.91E-04 (0.12)↓/↑	NS
*Chloroplast protease (Chp)*	VII	Chp_SNP4844	*C/T*	51/49	NS	0.0055 (0.08)^d^	NS
*Avr9/Cf-9 rapidly elicited protein 20 (ACRE20)*	X	ACRE20_SNP7156	*G/C*	82/18	0.0022 (0.08)↓/↑	NS	NS
*Magnesium-protoporphyrin IX monomethyl ester [oxidative] cyclase, chloroplast (MPP)*	X	MPP_SNP2580	*G/C*	68/32	0.0025 (0.09)↓/↑	0.001 (0.10)↓/↑	NS

a *Chr. = Chromosome*.

b *NS = not significant (P > 0.01)*.

c *Marker effect is shown by the arrow, ↑: positive effect (greater resistance, earlier maturity); ↓: negative effect (greater susceptibility, later maturity)*.

d *the direction of the allele effect was inconsistent between genotype classes*.

### Linkage analysis

**Figure 8:**
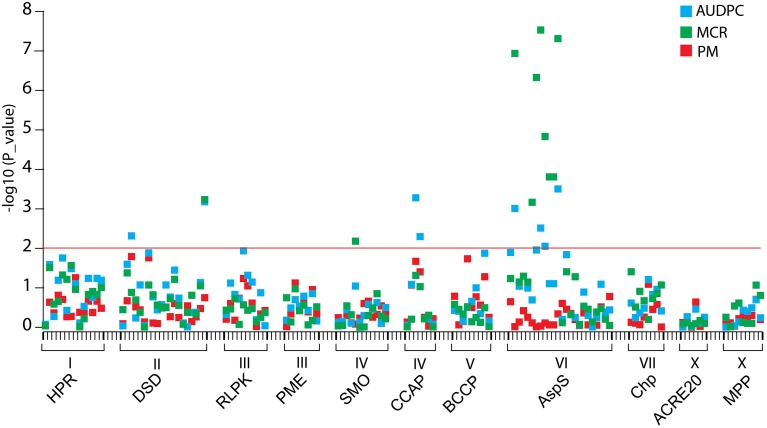
**Linkage plot for SNPs from 11 candidate loci**. SNPs are plotted on the x-axis and the –log_10_ of the *p*-values on the y-axis. The traits AUDPC, MCR, and PM are shown by blue, green, and red squares, respectively. The threshold *p*-value *p* = 0.01 is indicated by the horizontal line. Gene acronyms and chromosome positions are shown below the x-axis.

The half-sib families SL1 and SL2 and their three parents were genotyped for 127 SNP markers at 11 candidate loci by amplicon sequencing (118 SNPs) or pyrosequencing (9 SNPs). Linkage analysis with AUDPC, MCR and PM in the combined SL families identified 13 SNPs (*p* < 0.01) at the loci *DSD, SMO, CCAP*, and *AspS* that were linked with QTL for MCR or AUDPC or both (Figure 8, Table 6). None of the SNPs was linked with QTL for PM. DSD_SNP1516 was the only SNP that showed linkage as well as association with resistance to late blight (Tables 5, 6). The SNPs CCAP_SNP9779 and CCAP_SNP9786 virtually co-segregated, the slightly different allele frequencies most likely resulting from few errors in scoring the allele dosage in tetraploid heterozygous genotypes. These two SNPs were also present in the PIN184 population. Similarly, the SNPs AspS_SNP6253, AspS_SNP6260, and AspS_SNP6303 co-segregated in the SL families and were also present in the PIN184 population, showing LD with each other (Supplemental File S8). The haplotype *AspS_C*_6253_*T*_6260_*G*_6303_ was inherited from the susceptible parent Phy20. The remaining five SNPs in the *AspS* gene tagged a major QTL for MCR in the SL families, explaining up to 37% of the variation. The SNP alleles linked with greater resistance all descended from both resistant parents Phy14 and Phy16, in which they were present in variable allele dosage. These five SNPs were very rare (MAF < 1%) in the PIN184 population and were therefore excluded in the association analysis. One linked SNP (AspS_SNP6303) in the *AspS* locus caused the conservative amino acid substitution arginine/lysine (Supplemental File S5D).

**Table 6:** **SNPs linked with resistance to late blight in the combined families SL1 and SL2 (***n*** = 111), markers, allele frequencies, and allele effects**.

**Locus**	**Chr**.	**Marker**	**Allele**	**Phy14 genotype**	**Phy16 genotype**	**Phy20 genotype**	**Allele Frequency (%)**	**MCR *P*-value (*R*^2^)**	**AUDPC *P*-value (*R*^2^)**
*Delta (7)-sterol-C5 (6)-desaturase (DSD)*	II	DSD_SNP_pyro3220	*A/C*	*AAAC*	*ACCC*	*AACC*	54/45	0.0006(0.16)^*^	0.0007(0.16)^*^
		DSD_SNP1516	*T/G*	*TTTG*	*TTGG*	*TTGG*	61/39	NS	0.0048 (0.11)^*^
*Squalene monooxygenase (SMO)*	IV	SMO_SNP0177	*A/G*	*AAAA*	*AAAA*	*AAAG*	88/13	0.007 (0.07)↑/↓	NS
*Clathrin coat assembly protein AP17 (CCAP)*	IV	CCAP_SNP9779	*T/A*	*TTTT*	*TTTT*	*TTTA*	85/15	NS	0.0005 (0.17)^*^
		CCAP_SNP9786	*T/A*	*TTTT*	*TTTT*	*TTTA*	81/19	NS	0.0050 (0.13)^*^
*Asparagine synthetase (AspS)*	VI	AspS_SNP6253	*C/T*	*TTTT*	*TTTT*	*CCCT*	39/61	0.0001 (0.17)^*^	NS
		AspS_SNP6260	*T/G*	*GGGG*	*GGGG*	*TTTG*	39/61	0.0001 (0.17)^*^	NS
		AspS_SNP6303	*G/A*	*AAAA*	*AAAA*	*GGGA*	36/64	1.47E-05(0.21)^*^	0.0089 (0.10)^*^
		AspS_SNP5079	*C/A*	*CCCA*	*CCCA*	*CCCC*	89/11	4.87E-08 (0.27)↑/↓	0.0003 (0.14)↑/↓
		AspS_SNP6316	*G/T*	*GGTT*	*GGTT*	*GGGG*	80/20	2.95E-08 (0.29)↑/↓	0.0031 (0.12)^*^
		AspS_SNP6399	*T/G*	*TTTG*	*TTGG*	*TTTT*	78/22	4.70E-07 (0.33)↑/↓	NS
		AspS_SNP6404	*G/A*	*GGGA*	*AAAA*	*GGGG*	62/38	0.0007 (0.19)^*^	NS
		AspS_SNP6431	*A/G*	*AAAG*	*AGGG*	*AAAA*	73/27	1.16E-07 (0.37)↑/↓	0.001 (0.19)

Chr. = Chromosome; Marker effect shown by an arrow, ↑ shows positive effect (greater resistance); ↓ shows negative effect (greater susceptibility); NS = not significant (P > 0.01);

* *the direction of the allele effect was inconsistent between genotype classes*.

## Discussion

The analysis of gene expression, either in response to pathogen infection or between pathogen resistant and susceptible plants, is a long standing molecular genetic approach to discover and functionally characterize genes that play a role in host pathogen interactions (Chappell and Hahlbrock, 1984; Somssich et al., 1986; Fritzemeier et al., 1987). In recent years, new technology based on high throughput sequencing has dramatically scaled up the detection of genes that show differential expression in potato plants with different levels of resistance to late blight (Ros et al., 2005; Lindqvist-Kreuze et al., 2010; Gyetvai et al., 2012; Draffehn et al., 2013). The cultivated potato is a polyploid, non-inbred, highly heterozygous species and harbors a tremendous amount of DNA variation (Rickert et al., 2003). Differential transcript levels can therefore arise from background DNA variation between heterozygous genotypes that has nothing to do with resistance. Experiments such as gene silencing and mutant complementation analysis are required to ultimately confirm a causal role of a differentially expressed gene in resistance. The limitation in the number of genes that can be functionally characterized in this way requires the selection of the most promising candidates. Starting with 266 361 unique 26 bp sequence tags counted in nine genotype pools by sequencing nine SuperSAGE libraries, we filtered out 720 potato genes with apparently different transcript levels before and after controlled infection with *P. infestans* of plants that contrasted for maturity corrected resistance (MCR) as well as genotype at the *StAOS2* locus. The principle of bulked segregant analysis (Michelmore et al., 1991; Kloosterman et al., 2010) was used to homogenize, at least to some degree, the genetic background (Draffehn et al., 2013). The selection of the 22 genes described in this study from 720 candidate genes was by no means comprehensive or exclusive. Our selection was biased against multigene families with high sequence similarity such as chlorophyll a/b binding proteins, due to the technical difficulty of developing gene specific primers, against “ordinary,” well known genes such as ribosomal genes or known pathogenesis related genes, and (with one exception) against genes with unknown function, although genes of this type were frequent among the 720 candidates and such criteria do not disqualify a gene for a functional role in quantitative resistance. The selected genes had sufficiently distinct sequences for gene specific primer design, the frequency of the same unique SuperSAGE tag was consistently different in resistant and susceptible genotype pools before as well as after infection with *P. infestans* (Figure 1, Supplemental File S4), most of them co-localized with mapped QTL for late blight resistance (Figure 2) and their annotation suggested roles in defense responses (*ACRE20, UPA18, Kiw*), signaling and regulation (*SABP, MADS, RLPK*), chloroplast processes (*PSP, Chp, MPP*), cell wall modification (*Agp, PME*), protein biosynthesis, degradation, transport, and conformation protection (*EIF, Pubq, CCAP, SLP, HSP70*), lipid metabolism (*BCCP*), sterol biosynthesis (*SMO, DSD*), photorespiration (*HPR*), and nitrogen/amino acid metabolism (*AspS*). The selected genes were evaluated first for reproducibility of differential expression in genotype pools assembled in a way slightly different than the ones used in SuperSAGE and in independent infection experiments with *P. infestans*, and subsequently for association of SNPs at corresponding genomic loci with resistance to late blight and plant maturity.

### Reproducibility of resistance assessments and differential expression of candidate genes

Kinetics and quantity of the defense response of potato plants to infection by *P. infestans* vary considerably between independent infection experiments, despite the control of light, temperature, humidity and sampling times (Gyetvai et al., 2012; Draffehn et al., 2013). This is likely due to natural variation of the physiological state of the test plants and of the infectivity of the inoculum, which are difficult to standardize precisely from experiment to experiment. Reproducibility also depends on the phenotypic resistance assessment of the test genotypes, particularly when analyzing quantitative resistance. Three very different methods of resistance quantification, (i) evaluation of MCR in the field, (ii) transcript level of a *P. infestans* housekeeping gene, and (iii) AUDPC, the latter two evaluated in a growth chamber, showed remarkable consistency in sorting 24 SL genotypes into two groups with higher and lower late blight resistance levels (Supplemental File S1). In particular the consistency of the ranking of genotypes by MCR and by transcript level of the *P. infestans* ribosomal gene (only one genotype switched from the susceptible to the resistant group in Pools_2) confirms the observation that *P. infestans* growth measured by qRT-PCR during the first 3 days after controlled infection, before disease symptoms become visible, is an indicator for field resistance (Draffehn et al., 2013).

Reproducibility of differential expression was found to be overall better when assessed with pyrosequencing compared with qRT-PCR. In pyrosequencing, a DNA fragment of about 100–300 base pairs is first amplified by standard PCR, then a small internal portion (20–30 bp) containing the target SNP is sequenced from a single strand template using a third sequencing primer. These further steps could increase the specificity of pyrosequencing compared to qRT-PCR. As stated by Draffehn et al. (2013), sensitivity and specificity of qRT-PCR might be insufficient for reliable detection of quantitative differences between low expressed, allele specific transcripts detected by SuperSAGE.

When tested in two or four pairs of genotype pools with contrasting resistance to late blight (R and S pools), at three infection time points and in three independent infection experiments, the SuperSAGE expression pattern of nine genes (*ACRE20, Pubq, SLP, PME, SMO, DSD, MPP, HPR, UPA18*) was reproducible in more than 70 percent of all comparisons between R and S genotype pools and that of four genes (*CCAP, HSP70, BCCP, MADS*) was reproducible in 60 to 70 percent (Tables 3, 4). These 13 genes are considered as validated candidates for having a functional role in quantitative resistance to late blight.

### Putative functions of validated candidate genes

*SMO* and *PME* were expressed at higher level in S compared with R genotype pools, suggesting a role in susceptibility to *P. infestans*. Plant susceptibility genes enable pathogen entry into the host and facilitate nutrient provision (Hückelhoven et al., 2013; Lapin and Van den Ackerveken, 2013). *PME* was up regulated upon infection while *SMO* was down regulated. Squalene monooxygenase or squalene epoxidase (*SMO*) is a key enzyme in the biosynthesis of phytosterols, important membrane components and precursors of brassinosteroid plant hormones (Piironen et al., 2000). Pectin methylesterases (PME's) demethylate pectins, major structural components of plant cell walls with a key role in protection against environmental stresses and pathogen attacks (Pelloux et al., 2007). The action of PME's renders pectin susceptible to hydrolysis by pathogen enzymes such as endopolygalacturonases (An et al., 2008). Our results are in line with the findings in other plants. Ma et al. (2013) showed an increase in *PME* expression in susceptible but not in resistant banana cultivars after wounding and attack by *Fusarium oxysporum* f.sp. *cubense*. Over expression of a PME-inhibitor in Arabidopsis reduced PME activity and expression level which resulted in higher resistance to *Botrytis cinerea* (Lionetti et al., 2007).

*ACRE20, Pubq, SLP, CCAP*, and *HSP70* were expressed at higher level in R compared with S genotype pools. The action of these genes might increase late blight resistance. The *Avr9/Cf-9* rapidly elicited gene 20 (*ACRE20*) of tobacco encodes a putative EF-hand calcium binding protein (Day et al., 2002), which was elicited by the avirulence peptide Avr9 recognized by the tomato *Cf-9* gene for resistance to the fungal pathogen *Cladosporium fulvum* (Rowland et al., 2005). Expression of the potato *ACRE20* homolog was transiently upregulated upon infection in SuperSAGE (Supplemental File S4) but was consistently down regulated in all four sets of genotype pools in this study (Figure 4, Table 3). Such contradictory observations may be explained by the natural variation of infection kinetics in combination with slightly different time windows for sampling (T0, T1, T2 in SuperSAGE, T0, T2, T3 in this study). Polyubiquitin (*Pubq*) is post-translationally attached to other proteins (ubiquitination), which among other processes, targets them for degradation by the 26S proteasome (Li and Ye, 2008; Trujillo and Shirasu, 2010). Protein ubiquitination and degradation by the 26S proteasome play an important regulatory role in several plant defense signaling pathways (Trujillo and Shirasu, 2010; Marino et al., 2013). Subtilisin-like proteases (SLPs) are involved in plant development and signaling cascades. They accumulate in the extracellular matrix where the first contact occurs between host and pathogen, suggesting a role in pathogen recognition, defense signaling and priming (Figueiredo et al., 2014). The clathrin coat assembly protein AP17 (*CCAP*) was first described in maize where it is associated with the plasma membrane (Roca et al., 1998). A role in pathogen resistance has not been described so far. Clathrin-coated vesicles are involved in intracellular protein trafficking and endocytosis (Beevers, 1996). Heat shock cognate 70 kD protein (*HSP70*) is a molecular chaperone that is induced in response not only to abiotic stress (Wang et al., 2004) but also to fungal elicitor (Chivasa et al., 2006).

*UPA18, DSD, MPP, HPR, MADS*, and *BCCP* showed contrasting expression of two SNP alleles in R and S genotype pools. In most cases, the observed differential expression seemed to be in part or on the whole the consequence of different allele frequencies in R and S genotype pools, suggesting that allelic variation at these loci is linked with increased or decreased resistance to late blight. The exception was *BCCP*, where the differential expression of the “resistant” allele was consistently observed in Pools_4 in the absence the “susceptible” allele. Pools_4 were assembled based on MCR plus the genotype at the *StAOS2* locus, whereas the other R and S pools were mixtures with respect to *StAOS2* genotype. Possibly as a consequence, the expression patterns of Pools_4 were most distinct from Pools_1, _2, and _3 (Figure 5). The observed *BCCP* expression pattern might result not only from the contrasting MCR levels but also from the contrasting *StAOS2* genotype of Pools_4. Both genes play important roles in fatty acid and secondary metabolism in the chloroplast. Based on sequence homology, *BCCP* encodes Biotin carboxyl carrier protein, a subunit of the heteromeric acetyl-coenzyme A carboxylase (ACCase) complex, which catalyses the first committed step of fatty acid biosynthesis in chloroplasts (Nikolau et al., 2003; Sasaki and Nagano, 2004). Unsaturated fatty acids are the precursors of the jasmonate pathway, of which the first steps also take place in the chloroplast. Allelic variation of *StAOS2* could indirectly alter *BCCP* transcript levels via an unknown number of intermediate steps, for example via variable jasmonate levels (Draffehn et al., 2013). Delta(7)-sterol-C5(6)-desaturase (*DSD*) functions downstream of *SMO* in sterol biosynthesis. Sterols are crucial lipid components that regulate membrane permeability and fluidity and are the precursors of bioactive steroids (Silvestro et al., 2013). The Arabidopsis mutant alleles *dwf7* and *STE1*of this gene cause a dwarf phenotype (Choe et al., 1999). The chloroplastic Magnesium protoporphyrin IX monomethyl ester [oxidative] cyclase (*MPP*) is involved in chlorophyll biosynthesis (Peter et al., 2010) and in absicic acid signaling (Wu et al., 2009). *MADS* is one member of the superfamily of MADS-box transcription factors, which regulate multiple plant developmental processes (Smaczniak et al., 2012). This particular gene might regulate potato quantitative defense responses to *P. infestans*. Hydroxypyruvate reductase (*HPR*) is part of the photorespiratory cycle, one of the major pathways of plant primary metabolism which also plays a role in defense responses (Rojas et al., 2014; Timm et al., 2008). UPA18 (UPregulated by AvrBs3) is a pepper gene of unknown function, which was induced within 8 h by the AvrBs3 effector of *Xanthomonas campestris* pv*. vesicatoria* (Xcv) upon infection (Kay et al., 2007, 2009). The potato homolog showed no upregulation 2 and 3 days after infection with *P. infestans*, but allelic differential expression in R and S pools was highly reproducible. The SNP defining the two *UPA18* alleles led to a non-conservative substitution of isoleucine by methionine, which can modify protein conformation, function, or posttranslational modification.

### Associations between SNPs at candidate loci and resistance to late blight

Differential transcript levels ultimately result from DNA variation directly in the observed gene or indirectly in other genes that control the expression of the observed gene. Epigenetic DNA modification may also cause differential gene expression. Only in the first case we can expect to find an association between DNA variation at the candidate locus and phenotypic variation such as maturity corrected resistance to late blight. *Pubq* might be an example for the second or third possibility. Higher transcript level of this gene in R vs. S genotype pools was among the most reproducible expression patterns. However, DNA variation in this gene was exceptionally low, such that association analysis was not possible. Association mapping of SNPs in 11 candidate genes, nine showing reproducible (>60%) differential expression in R and S genotype pools (*ACRE20, BCCP, CCAP, DSD, HPR, MPP, PME, SMO, UPA18*) and three with lower reproducibility (< 60%) (*AspS, Chp, RLPK*) yielded nine SNPs in seven genes (*ACRE20, BCCP, Chp, DSD, HPR, MPP, PME*) which were associated with rAUDPC and/or MCR but not with plant maturity. Only one SNP in the *CCAP* gene showed association with PM (Table 5). The total variance explained by a single SNP ranged from 6 to 13%, indicating small to moderate effects on resistance. Two SNPs in *BCCP* forming a haplotype showed the strongest association with MCR. Both SNPs caused the non-conservative amino acid substitution of proline by serine (Supplemental File 5c), which could affect the conformation and/or enzymatic properties of the BCCP protein and thereby the function of the ACCase complex. Although, reproducibility of the expression pattern of chloroplast protease (*Chp*) was only 50%, one SNP in this gene was associated with rAUDPC. The tomato and Arabidopsis homologs of *Chp* are annotated as ATP-dependent, thylakoid-bound Zn protease (*FtsH*) that is important in the biogenesis and maintenance of photosystem II (Chi et al., 2012).

All seven associated genes are located on different chromosomes or chromosome arms (Figure 2). Accordingly, no LD was observed between associated SNPs at different loci. The SNPs associated with MCR and/or rAUDPC represent novel diagnostic markers for resistance to late blight and are a valuable extension of the few diagnostic markers available so far (Gebhardt et al., 2004; Pajerowska-Mukhtar et al., 2009; Odeny et al., 2010). Except *Chp*, all SNP alleles associated with increased resistance to late blight were the minor frequency alleles in the PIN184 population (Table 5). Increasing collectively the frequency of these alleles in breeding populations, while preserving other agronomic qualities such as yield and culinary qualities by phenotypic selection, might result in new varieties with increased field resistance to late blight that is not compromised by late maturity.

### Novel, rare resistance allele(s) for MCR identified by linkage mapping

SNPs in the same genes as analyzed for association were tested for linkage with AUDPC, PM, and MCR in the combined half-sib families SL1 and SL2 (*n* = 111), as the size of each family alone was too small for QTL mapping. Linkages with resistance alleles inherited from either Phy14 or Phy16, the seed parents of family SL1 and SL2, respectively, were probably not detectable. Nevertheless, 13 SNPs in four genes, eight of those in the *AspS* gene, showed linkage with MCR and/or AUDPC and none was linked with PM (Figure 8, Table 6). There was very little overlap between association and linkage mapping. Only one SNP, DSD_SNP1516 for which all three parents of the SL families were heterozygous, was linked as well as associated with resistance to late blight, indicating that it represents a common variant. DSD was also the only candidate gene, where the SNP distinguishing the allelic SuperSAGE tags with contrasting expression was linked with MCR. Taken together, expression profile and genetic analysis make *DSD* one of the most promising candidate genes for playing a direct role in maturity corrected resistance to late blight.

The largest effect on MCR was detected by five SNPs in the *AspS* gene (AspS_SNP5079, AspS_SNP6316, AspS_SNP6399, AspS_SNP6404, and AspS_SNP6431), which were inherited from both resistant parents of the SL families but were very rare in the PIN184 association panel. This indicates that one or more rare resistance alleles for MCR segregated in the SL families. In the case of rare alleles, linkage mapping in experimental populations is superior over association mapping, where rare alleles are discarded or do not reach statistical significance (Simko et al., 2007). This new MCR allele(s) can be introgressed into other cultivars using the linked SNPs. Three additional, co-segregating SNPs (AspS_SNP6253, AspS_SNP6260, and AspS_SNP6303) tagged a different MCR allele descended from the susceptible parent Phy20. These SNPs were common in the PIN184 population and were still in significant LD but did not show association with late blight resistance. *AspS* was one of the candidate genes with contrasting allelic expression, which can result from different allele frequency in R and S genotype pools. Different allele frequencies are not restricted to the gene that is causal for the phenotypic effect but can extend to physically closely linked genes, which share the same haplotype block with the causal gene. The effects detected by SNPs in the *AspS* gene might therefore result from LD with the causal gene. Nevertheless, asparagine synthetase (*AspS*) is an interesting candidate gene based on functional analysis in other plant species. AspS is a key enzyme in the biosynthesis of asparagine and is required for nitrogen assimilation and defense responses against microbial pathogens. Hwang et al. (2011) reported that *Capsicum annuum* asparagine synthetase1 (*CaAS1*) was rapidly induced in leaves upon infection with *Xanthomonas campestris* pv*. vesicatoria* (Xcv). Silencing *CaAS1*in pepper plants resulted in enhanced susceptibility to Xcv. Arabidopsis plants overexpressing *CaAS1* exhibited enhanced resistance to *Pseudomonas syringae* pv*. tomato* DC3000 and to the oomycete pathogen *Hyaloperonospora arabidopsidis*. *AspS* might be involved in induction of reactive oxygen species (ROS) and nitric oxide (NO) (Hwang et al., 2011). ROS and NO are important signaling molecules in the complex signaling pathways controlling disease resistance (Boller and Keen, 2000).

## Conclusions

1. Extensive expression studies of 22 candidate genes in various genotype pools with contrasting levels of quantitative resistance to *P. infestans* and in independent infection experiments, either by qRT-PCR or pyrosequencing, validated 13 genes (59%) as novel candidates for playing a functional role in resistance to late blight not compromised by late maturity. Gene silencing by targeted genome editing using new technology like CRISPR/Cas9 (Sander and Joung, 2014) and complementation analysis in mutants of *Arabidopsis thaliana* (Pajerowska-Mukhtar et al., 2008) are required for confirming their function in quantitative resistance.
2. The expression profiles of the validated candidate genes suggest that part of maturity corrected resistance to late blight is a constitutive plant state, manifest prior to infestation by *P. infestans*. Their putative functions suggest that processes in the chloroplast such as fatty acid biosynthesis, photosynthesis, and photorespiration play a role in MCR, together with phytosterols, membrane and cell wall modifications, protein modification, transport and degradation, and pathogen elicitor and stress responsive genes.
3. Association mapping of SNPs in 11 candidate genes identified seven SNPs in five differentially expressed genes that were associated with maturity corrected resistance to late blight. These SNPs are novel diagnostic markers to be used in resistance breeding. Alleles of genes *ACRE20, BCCP, DSD, MPP*, and *PME* are good candidates for contributing directly to the natural variation of MCR. To confirm this, comparative functional characterization of alleles associated with greater resistance or susceptibility is required, for example by quantitative complementation analysis (Pajerowska-Mukhtar et al., 2008) or biochemical studies in heterologous systems (Fridman et al., 2004).
4. Linkage mapping of SNPs in 11 candidate genes identified five SNPs in the *AspS* gene, which tagged one or more rare alleles for maturity corrected resistance to late blight segregating in the SL families. These SNPs are useful for introgressing the novel resistance allele(s) into cultivars. Whether the *AspS* locus itself is responsible for the resistance phenotype or another, physically closely linked gene requires further studies.

### Conflict of interest statement

The authors declare that the research was conducted in the absence of any commercial or financial relationships that could be construed as a potential conflict of interest.

## Supplementary material

Supplemental File S1 **Pools**.

Supplemental File S2 **Primers**.

Supplemental File S3 **241_SolCAP_SNPs**.

Supplemental File S4 **SuperSAGE tags**.

Supplemental File S5 **Amino acid sequences**.

Supplemental File S6 **Pyrosequencing**.

Supplemental File S7 **Candidate_genes_sequences_and_SNPs**.

Supplemental File S8 **LDmatrix**.
