# Supplementary material for: Selection and validation of potato candidate genes for maturity corrected resistance to Phytophthora infestans based on differential expression combined with SNP association and linkage mapping
Source: Front Genet. 2015 Sep 23;6:294. doi: 10.3389/fgene.2015.00294 (PMC4585299; doi:10.3389/fgene.2015.00294)
Supplement: Supplemental File S1 — Pools. [file DataSheet1.DOCX]

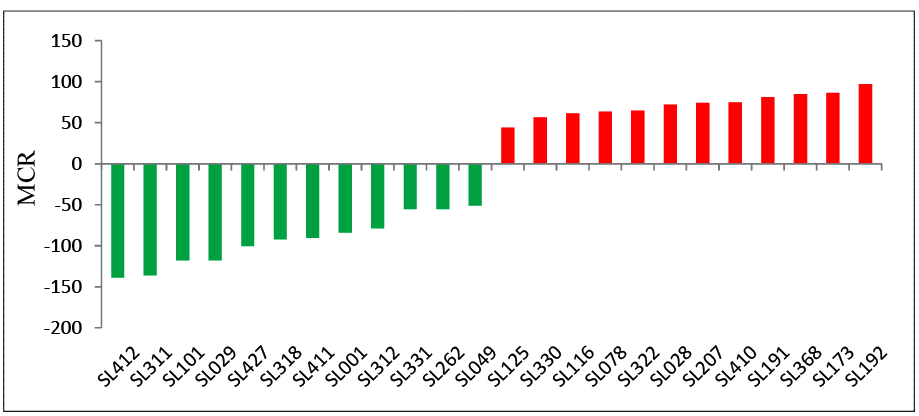

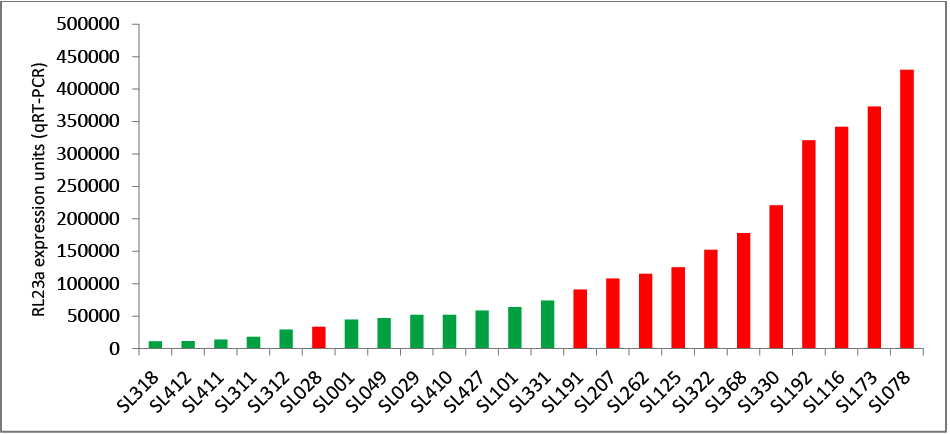


**A**

**B**


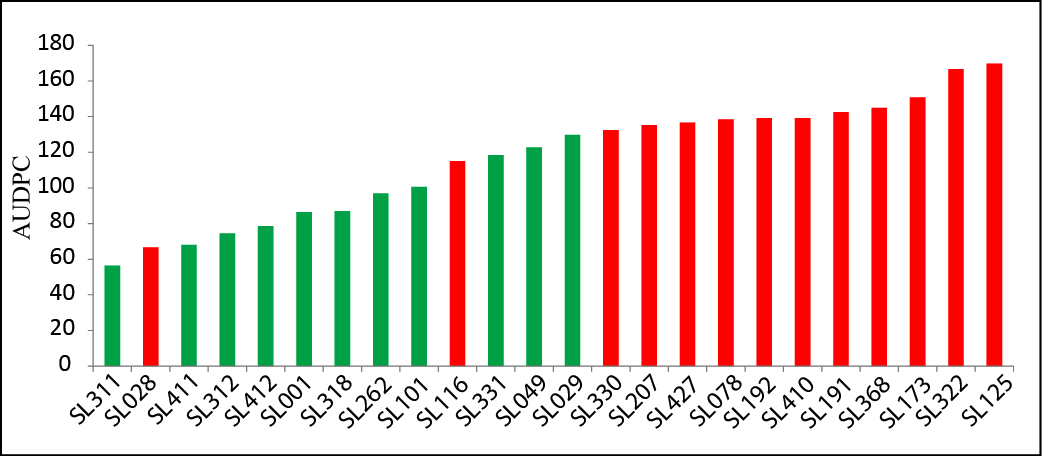

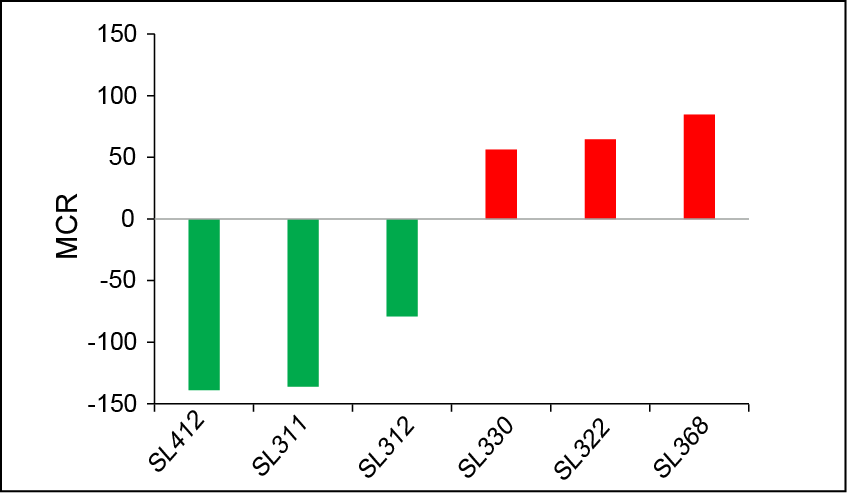


**C**

**D**

**Supplemental File S1**. Ranking of 24 SL genotypes used to construct RNA pools according to resistance phenotype as determined by three methods. (**A**) MCR according to the 2010 field evaluation. Pools_1 were constructed by pooling equal amounts of RNA from the 12 most resistant (green bars) and susceptible (red bars) genotypes. Negative MCR values indicate higher resistance. (**B**) *P. infestans* growth quantified by qRT-PCR of the *Phytophthora* transcript RL23a. Bars represent the average expression units of T2 and T3 of three infection experiments. Bars are colored as in (**A**). Pools_2 were constructed by pooling equal amounts of RNA from the six most resistant (the first 6 genotypes to the left) and susceptible (the last 6 genotypes to the right) genotypes. (**C**) AUDPC evaluated on the 3^rd^ compound leaf from day three until day five after inoculation. Bars represent the average AUDPC values of three infection experiments. Bars are colored as in (A). Pools_3 were constructed by pooling equal amounts of RNA from the five most resistant (the first 5 genotypes to the left) and susceptible (the last 5 genotypes to the right) genotypes. (**D**) Subset of genotypes in (**A**) with contrasting MCR in the field 2010 plus contrasting genotype at the *StAOS2* locus. The three genotypes to the left (green bars) were homozygous for haplotype *StAOS2_A_691_C_692_* while the three genotypes to the right (red bars) had three copies of haplotype *StAOS2_G_691_G_692_* and one copy of haplotype *StAOS2_A_691_C_692._* Pools_4 were constructed by pooling equal amounts of RNA from three resistant (green bars) and susceptible (red bars) genotypes.
