# Supplementary material for: Selection and validation of potato candidate genes for maturity corrected resistance to Phytophthora infestans based on differential expression combined with SNP association and linkage mapping
Source: Front Genet. 2015 Sep 23;6:294. doi: 10.3389/fgene.2015.00294 (PMC4585299; doi:10.3389/fgene.2015.00294)
Supplement: Supplemental File S2 — Primers. [file DataSheet2.DOCX]

**Supplemental File S2**. PCR information for 22 candidate genes: Primer sequences, amplicon size, annealing temperature (Ta).

| **Gene annotation (acronym)** | **Method** | **Primers 5' - 3' ^1^** | **Amplicon size [bp]** | **Ta** |  |
| --- | --- | --- | --- | --- | --- |
| Arabinogalactan protein (Agp) | qRT-PCR | F: CTACCCTATTCGTTCCTACCGT  R: CCTCAAACTCCTCATCTCAATG | 202 | 61 |  |
| Eukaryotic translation initiation factor 4e type (EIF) | qRT-PCR | F: GATGCTTCCTTGCGCGAC  R: CATACACATCAGAAGAACTACAGGC | 245 | 60 |  |
| Avr9/Cf-9 rapidly elicited protein 20 (ACRE20) | qRT-PCR | F: AGCCCTTTACACAGCTCGTC  R: AGTTGAATTAATGGAGAAACAGCTT | 157 | 58 |  |
|  | Amplicon sequencing | F: GAACCACGGGCCGACATATT  R: ATCGAGATAAGAGCAGAACCGC | 1218 | 62 |  |
| Polyubiquitin (Pubq) | qRT-PCR | F: CTTCTGAATGTCCTGTGTCTG  R: GCAGCAAAAACAAAAGAAC | 140 | 54 |  |
| Clathrin coat assembly protein AP17 (CCAP) | qRT-PCR | F: CTGCACTTCTCTTCCGGCTG  R: GCCTCTGGAGTTGACAAAAGA | 212 | 53 |  |
|  | Amplicon sequencing | F: ttctctggcacctcagttctg  R: TTGAATCCCCCACCACTCC | 866 | 61 |  |
| Subtilisin-like protease (SLP) | qRT-PCR | F: GGAAGTGCCAAAGAATACCA  R: TCCCCAGGGAAGTATAATG | 217 | 55 |  |
| Conserved gene of unknown function (Cgu) | qRT-PCR | F: GTTATCCACCTCCTGGC  R: AACTTGGCTGCAGGATG | 256 | 54 |  |
| Heat shock cognate 70 kDa protein (HSP70) | qRT-PCR | F: TCCAATTATTGCCAAGATGTACCA  R: TCAAGCCAAAATACGCAAAAGT | 243 | 60 |  |
| Kiwellin (Kiw) | qRT-PCR | F: CTTGGGACTATTGCGATGGT  R: AGTTGAGCGGGCATTGAT | 217 | 58 |  |
| Photosystem II core complex proteins (PSP) | qRT-PCR | F: CCTGCTTTGCTTTGGGTAG  R: AATCATATGGCCAAACGC | 250 | 57 |  |
| Salicylic acid-binding protein (SABP) | qRT-PCR | F: GGTACTGATCATACGGCA  R: TCTCATCACACATTCTTCAA | 189 | 52 |  |
| Chloroplast protease (Chp) | qRT-PCR | F: CAAAGCAAGGGAAACAACTCA  R: GACTGGAGATGAATTCCGTGCTAT | 289 | 60 |  |
|  | Amplicon sequencing | F: GCTCCACTGAAACCAGGTGT  R: ATTTACTGCAGTGGGGGCTC | 623 | 60 |  |
| Pectin methyl esterase (PME) | qRT-PCR | F: CGTGTTGACGAATTTCAGTGAT  R: GGCCAAGTATAAGCATAAATTCTC | 110 | 60 |  |
|  | Amplicon sequencing | F. GTAACTAATTTCATGCAGCCG  R: AGGGAAACTCTTCTGCACCG | 410 | 60 |  |
| Squalene monooxygenase (SMO) | qRT-PCR | F: GGCAAAACTAAGTGAACCG  R: AGTTTCAAGAGTTTAGTCCAAC | 242 | 53 |  |
|  | Amplicon sequencing | F: ACTAAGTGAACCGCAAGCCA  R: TATCTTTGGTGTTGGCCGCT | 581 | 61 |  |
|  | Amplicon sequencing | F: TGCAAGGAAGGATTTCAAGC  R: GCACATCGGCTTTGATTTTT | 117 | 59 |  |
| SAND | qRT-PCR | F: CTGCTTGGAGGAACAGACG  R: GCAAACAGGACCCCTGAATC | 163 | 58 |  |
| Delta (7)-sterol-C5 (6)-desaturase (DSD) | Pyrosequencing | F: CATACATGGTAAGGTGTGGC  R:[Btn]CAACGATGGAAACACGAGAC  S:ATGTAATGTGCATGATTTG | 215 | 57 |  |
|  | Amplicon sequencing | F: TAGCCCCTCTTCTTTTTCC  R: CATTTGGTCCATCCGTTTTC | 1040 | 57 |  |
|  | Amplicon sequencing | F: AGCTAGCTAATTCATGATGTCCAGT  R: CCACCAACGATGGAAACACG | 538 | 61 |  |
| Magnesium-protoporphyrin monomethyl ester [oxidative] cyclase, chloroplast (MPP) | Pyrosequencing | F: GGCTTCTGAGTTATTGGCTG  R:[Btn]CTGATAATACAAGGGAGTGTCC  S:CATGTTCATTGTTGTAAGTT | 221 | 51 |  |
|  | Amplicon sequencing | F: GGAAGATGTTTTGTCAAGTGC  R: ACAAACCGCACAACTGCA | 536 | 58 |  |
| Hydroxypyruvate reductase (HPR) | Pyrosequencing | F: [Btn]TGCCCGAGCATTGTGAATTCAA  R:CCAGATTGAGAGTGTGGGTAGCT  S: ATATGAGTAAAACTTGTGTC | 287 | 60 |  |
|  | Amplicon sequencing | F: TGCAGCAAGAAGAATTGTGG  R: ATAACGGGAGAATGGGATCAA | 1091 | 59 |  |
| Up-regulated by AvrBs3 (UPA18) | Pyrosequencing | F: CTCCCCCTCCTCCACGAC  R:[Btn]CAATCCTTCCCCCAATGTC  S:GAAGCGTAGTGGGAAAAT | 203 | 60 |  |
| Biotin carboxylase carrier protein (BCCP) | Pyrosequencing | F: [Btn]GTTGATGAACGAAATAGAGGCTG ^2^  R:TTACCCGAACGGTTCTATGGTTT ^2^  F: [Btn]AGGCCACATAAGGACACAACC ^3^  R: TCGTCTATTCCATCACACTACCA ^3^  S:TTCAGCAACAACCTCA | 125  413 | 61  60 |  |
|  | Amplicon sequencing | F: TGGGACGATCTGTTGCCTTC  R: AAAGTCATGAAATAGAGGTCTCGT | 840 | 59 |  |
| MADS-box transcription factor 16 (MADS) | Pyrosequencing | F: CCTCGAAGAGCTGAAACTGC  R:[Btn]AGGGAAGTTGCCCTTACTGA  S:CAAAGATGCATTTGAGG | 194 | 59 |  |
| Asparagine synthetase (AspS) | Pyrosequencing | F: [Btn]GGAACATTAGTGGTGCTCAAGAA  R:GGAAGGATCAGCACGTCTTTTAGA  S:GAATAAAAGATCAAAACCAT | 124 | 60 |  |
|  | Amplicon sequencing | F: CCAAAGTCCAGAAGGACACTCTAT  R: GCAAGCCTGACCGTTCCT | 552 | 60 |  |
|  | Amplicon sequencing | F: GTACTCTAGCTTCTAAGCCCC  R: CGGTTGATGACTGATGTCCCC | 830 | 59 |  |
|  | Amplicon sequencing | F: CGCCTCAGAACCAAAGTGTCAT  R: CATCGTGGACCGGATTGGAGT | 1053 | 60 |  |
| Receptor-like protein kinase (RLPK) | Pyrosequencing | F: GTACCCTGGAGAATCCTAAG  R:[Btn]GGACAGTCTCTCATATTGGAG  S:AAAAGTTTGTCATGAAACTA | 188 | 52 |  |
|  | Amplicon sequencing | F: CGATTCACATTTCGTACCATGCT  R: CGAAGATGGATTGGAATCACTAG | 1123 | 61 |  |

**^1^** F = forward primer; R = reverse primer; S = sequencing primer; [Btn] = the position of biotin; acronym of each candidate is shown in parenthesis

^2^ Primers used for pyrosequencing with cDNA.

^3^ Primers used for pyrosequencing with genomic DNA
