# Supplementary material for: Selection and validation of potato candidate genes for maturity corrected resistance to Phytophthora infestans based on differential expression combined with SNP association and linkage mapping
Source: Front Genet. 2015 Sep 23;6:294. doi: 10.3389/fgene.2015.00294 (PMC4585299; doi:10.3389/fgene.2015.00294)
Supplement: Supplemental File S5 — Amino acid sequences. [file DataSheet5.DOCX]

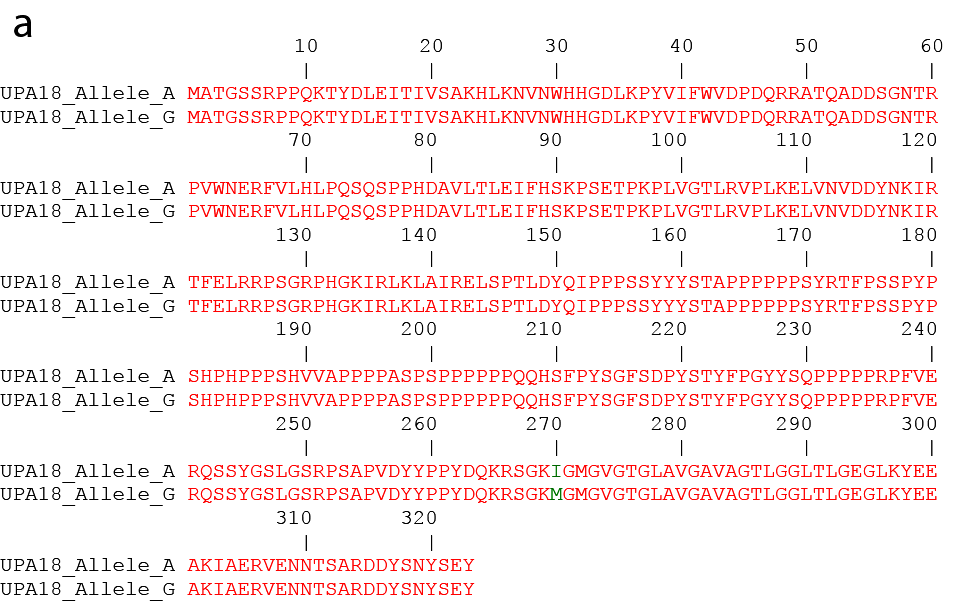


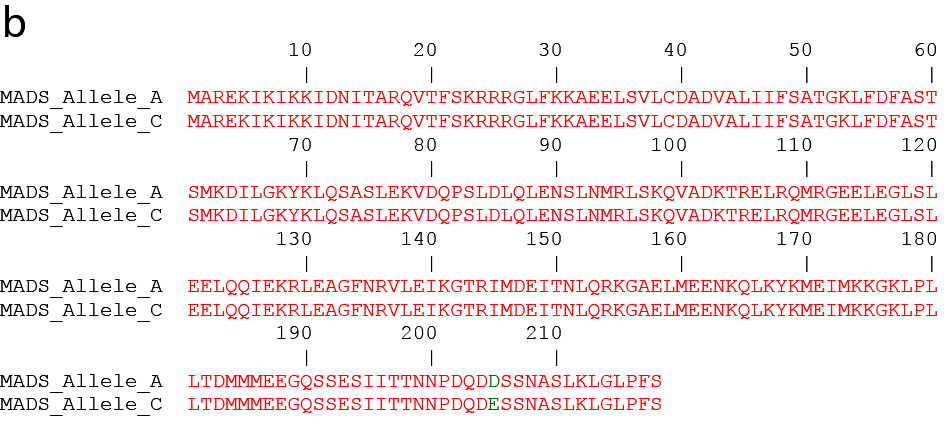


cd


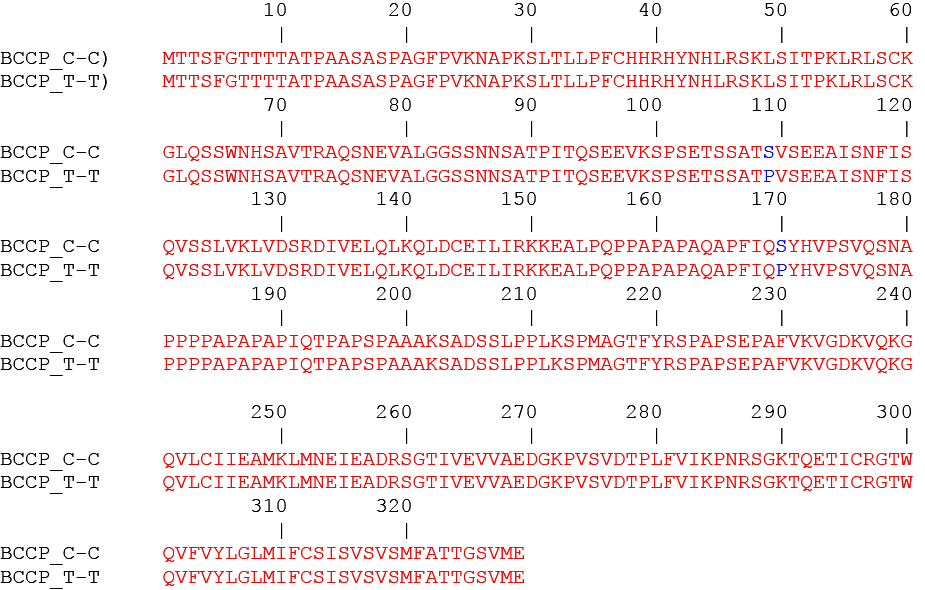


d

**
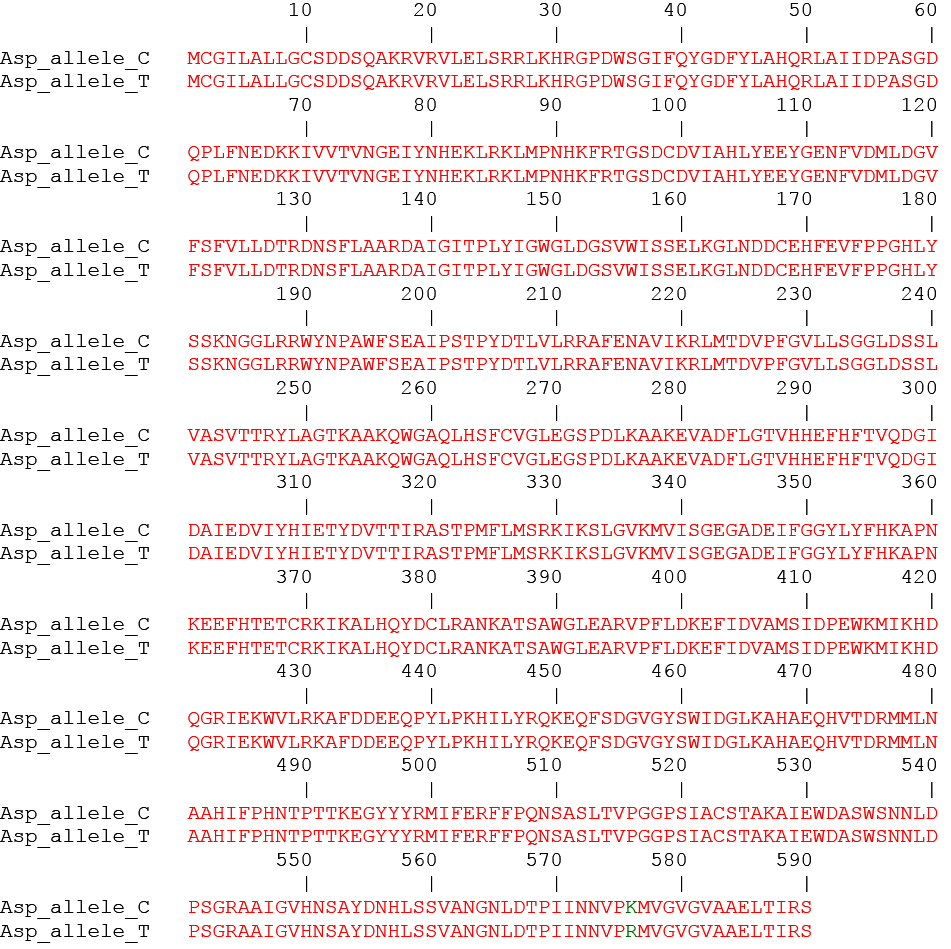
**

**Supplemental File S5.** Amino acid sequence alignments showing amino acid substitutions caused by (a) the SNP in the allelic tag of *UPA18,*  (b) the SNP in the allelic tag of *MADS*, (c) the two associated SNPs in *BCCP* and (d) the linked SNP (AspS_SNP6303) in *AspS*.
