# Supplementary material for: Selection and validation of potato candidate genes for maturity corrected resistance to Phytophthora infestans based on differential expression combined with SNP association and linkage mapping
Source: Front Genet. 2015 Sep 23;6:294. doi: 10.3389/fgene.2015.00294 (PMC4585299; doi:10.3389/fgene.2015.00294)
Supplement: Supplemental File S6 — Pyrosequencing. [file DataSheet6.DOCX]

**Supplemental File S6.** SNP allele specific expression analysis by pyrosequencing. The data represent the relative expression in percent (average of three infection experiments) at T0, T2 and T3 of the allele that was expressed at higher level in the resistant pool A2 in SuperSAGE (Supplemental File S4). Each SNP was tested on resistant (R) and susceptible (S) RNA pools in Pools_1, _2, _3 and _4.

|  |  | **Pools_1** | | **Pools_2** | | **Pools_3** | | **Pools_4** | |
| --- | --- | --- | --- | --- | --- | --- | --- | --- | --- |
| **Candidate gene** | **Time**  **points** | **R** | **S** | **R** | **S** | **R** | **S** | **R** | **S** |
| Delta (7)-sterol-C5 (6)-desaturase, C allele | T0 | 57.50 | 49.90 | 68.73 | 45.30 | 62.73 | 47.10 | 73.80 | 48.43 |
|  | T2 | 65.60 | 43.93 | 71.47 | 44.23 | 65.13 | 42.40 | 71.27 | 45.97 |
|  | T3 | 63.63 | 50.37 | 68.87 | 44.50 | 66.57 | 42.67 | 71.30 | 41.70 |
| Magnesium-protoporphyrin IX monomethyl ester [oxidative] cyclase, chloroplast (MPP), G allele | T0 | 24.37 | 22.17 | 30.20 | 16.87 | 37.60 | 20.83 | 45.63 | 26.67 |
|  | T2 | 31.33 | 21.43 | 39.00 | 16.20 | 34.80 | 24.13 | 39.23 | 22.67 |
|  | T3 | 31.60 | 22.87 | 45.87 | 11.00 | 38.03 | 26.53 | 50.97 | 23.77 |
| Hydroxypyruvate reductase (HPR), A allele | T0 | 21.63 | 12.60 | 17.23 | 9.50 | 17.43 | 16.93 | 18.50 | 11.63 |
|  | T2 | 17.80 | 10.23 | 18.70 | 14.60 | 18.90 | 12.97 | 21.33 | 13.47 |
|  | T3 | 19.33 | 9.37 | 20.57 | 12.80 | 23.83 | 11.87 | 27.13 | 15.70 |
| Up-regulated by AvrBs3 (UPA18), A allele | T0 | 62.20 | 55.83 | 65.17 | 62.10 | 70.33 | 64.73 | 72.13 | 70.03 |
|  | T2 | 61.20 | 54.07 | 69.37 | 55.50 | 72.27 | 60.40 | 74.37 | 65.83 |
|  | T3 | 56.33 | 49.80 | 68.53 | 60.87 | 70.53 | 59.93 | 71.80 | 64.47 |
| Biotin carboxylase carrier protein (BCCP), T allele | T0 | 64.47 | 63.40 | 55.47 | 63.53 | 64.37 | 64.67 | 80.00 | 64.67 |
|  | T2 | 61.30 | 60.13 | 56.30 | 62.60 | 64.37 | 61.17 | 77.90 | 61.60 |
|  | T3 | 61.03 | 56.80 | 53.47 | 57.23 | 61.80 | 59.30 | 78.73 | 59.83 |
| MADS-box transcription factor 16 (MADS), T allele | T0 | 50.23 | 49.23 | 48.23 | 49.17 | 52.83 | 49.47 | 49.83 | 54.00 |
|  | T2 | 50.20 | 51.63 | 52.20 | 50.37 | 51.67 | 51.23 | 55.73 | 47.90 |
|  | T3 | 49.60 | 47.63 | 54.67 | 51.57 | 54.10 | 51.40 | 52.50 | 51.17 |
| Asparagine synthetase (AspS), T allele | T0 | 79.17 | 58.53 | 78.43 | 55.07 | 59.10 | 71.97 | 89.23 | 97.60 |
|  | T2 | 77.73 | 66.80 | 86.40 | 71.07 | 63.50 | 79.77 | 88.87 | 90.37 |
|  | T3 | 78.60 | 69.37 | 90.30 | 69.87 | 69.00 | 81.20 | 88.40 | 97.70 |
| Receptor-like protein kinase_SNP1 (RLPK-1), T allele | T0 | 36.00 | 39.63 | 34.93 | 37.90 | 36.97 | 33.57 | 30.23 | 26.67 |
|  | T2 | 37.30 | 44.40 | 32.20 | 48.40 | 40.50 | 38.77 | 30.50 | 26.23 |
|  | T3 | 32.87 | 37.27 | 31.30 | 38.30 | 33.17 | 32.90 | 26.97 | 26.17 |
| Receptor-like protein kinase _SNP2 (RLPK-2), G allele | T0 | 80.23 | 78.60 | 82.30 | 78.50 | 76.73 | 82.23 | 79.37 | 83.93 |
|  | T2 | 78.90 | 76.10 | 82.17 | 75.87 | 73.87 | 81.43 | 80.43 | 83.73 |
|  | T3 | 83.43 | 80.33 | 82.97 | 80.30 | 81.13 | 82.33 | 84.30 | 82.10 |
