# Supplementary material for: Selection and validation of potato candidate genes for maturity corrected resistance to Phytophthora infestans based on differential expression combined with SNP association and linkage mapping
Source: Front Genet. 2015 Sep 23;6:294. doi: 10.3389/fgene.2015.00294 (PMC4585299; doi:10.3389/fgene.2015.00294)
Supplement: Supplemental File S7 — Candidate_genes_sequences_and_SNPs. [file DataSheet7.DOCX]

**Supplemental File S7**: Amplified sequences and SNP positions of eleven candidate genes. Primers for amplicon sequencing and pyrosequencing are highlighted green and blue, respectively. Primers used for sequencing are underlined. Exons are in red and introns in black letters. SNPs are identified with the last four digits (.****) of their position on the pseudomolecules of the DM genome sequence (version 4.03). Allelic SuperSAGE tag sequences are highlighted yellow.

Hydroxypyruvate reductase (HPR), PGSC0003DMG400006186

chr01:87614338..87616450

tgcagcaagaagaattgtggaggcagatgagttcatgagagctggcaaatatgaaggatggcttccacatttgta

.4437.4438

tgaaaatcttcgattctatttatt[G/C][T/C]gtcttctcaggtctataactgaattcttttggtctaaaagt

.4486 .4489

tcaatg[T/A]tt[T/G]cattgccaggtttgtggggaacttacttaaaggacaaacggttggcgtaattggggc

tggtcgtattggatctgcttatgctagaatgatggtaatcagttagtatatattatcctctaagtttgctttgaa

actaggttgccttgtgctttttgctctacatgttacaaaacttcatctttgctgaaactcatgaataagatattg

tgtgtaaatctgctatgttaattggagcaactttggtgttttatcgcaacaggttgaaggcttcaaaatgaactt

gatatactacgacctgtatcaatctactcgtctagaaaaattcgtgacaggcacgttttaatcagtctgctacat

tagcaaacttctcaagctcattgcatgtacttatgttttctcgcctttgcagcctatggtcagtttctgaaagcc

aatggtgaacaacctgtaacatggaaaagagcttcatccatggaggaggtcctccaagaggctgatgtggtaatt

atctacattgagagtgcacaattagatttcttattcacttatctgtatatacatatttattggattctcattcaa

.5114

gttcttatcttgattcttgatcaaaatgcttttctagataag[C/T]ctacatccaatccttgataaaacaac

.5141 .5144

[G/A]ta[C/T]catcttgtgaacaaagaacggctagcactgatgaagaaggtatatcatttgcaagaatatat

.5207 .5242 .5257 .5265

[C/T]attttgcttgctgatttactcaaaatgtgatat[C/A]ttgatattacttgg[C/T]ccaaact[G/T]

.5266 .5299 .5306

[T/G]taggaagcaatacttgtgaactgtagtagggg[G/C]cccgtg[G/A]ttgatgaagtagctcttgttga

gcatctgagggaaaatcccatgttccgtgttggccttgatgtctttgaggtaataacaatagtaatctgttcctt

tctgttgatcccattctcccgttatgaaggtgtaaaaggtgaatcccaaggtcaatgagatactgttgtgtcacg

cgccatttctaccaagcaaaaagctctgaaattaacctactcatgttgagtgtaggatgctttttccttgtcttg

tacattcaagtaatgctcttcttttataatcgtgttgtcttaggatgagccctacatgaaacccggtcttgcaga

catgaagaatgctgttgtggtgccccacatagcttctgcttctaaggtaattttgcacatatttatgtttttatc

tgtgcttaaccgtgtaaactaagaagcaatataatgttctgctatgacggctacttcccatctaattcgccaatt

atggtgatttttgatagtgttgccactttgtaagaaaaaaactaaaatctcctgattcttgatgcagtggactcg

cgagggaatggctacactggctgctctgaatgtcctggtacgcaattttttaaggacactgttgtgctaaaaatc

acagtgaacataaagacagacattgtattgtgtgtttggattctgaatgttgcatatgtttgtttacagggaaaa

attaaagggtatccaatttggagtaatccaaacagtgtggagccgttcttaaatgagaatgcaacgccccctgct

gcatgcccgagcattgtgaattcaaaagctataggtaattaagtcatatatgcaattcataagagctgcaaattt

ggttgagggattttgctaaatacataatggttaattattgcaggcttagctgtttcaaagctgtgaagttgaaat

gttttaagtgattcaacatatgtcagactattggaaggagttgataatgtttgctatactatatcatgtatctct

.6346(pyro)

catttgtgtatattggtttacatgattttgctataattagga[T/C]ggacacaagttttactcatattctcttg

tttaacacatacattcaacccggcccgcatgcgggttactaactccgcctgttagctacccacactctcaatctg

g

Delta (7)-sterol-C5 (6)-desaturase (DSD), PGSC0003DMG400026401

chr02:41420750..41423282

tagcccctcttctttttcctctccatcttctcactccatacttcctcctcttctggtggaccatcgccgccggag

tctggtaaaacccgacccgacccggatatatttctctttctccgagtataccgtcggagatggaggattacttga

agctgttcgtggaggagacatcgttttacaaccgtatggttttgggtacattcttgccggaatcatggtggggac

cacttcctcatatgcttcaaggatggctccgtaactacattggcggtgttttactttacttcatctccggtttcc

tctggtgcttctacatttatcacttgaaacgcaatgtctatattcccaaaggtatgattttttcccccctttttc

tccccttttttttttttgaatttcgtttgtgtatagatgatttctttgcattatttgaaaattataagttcatgg

aattaagttgactgttttagtaaagcttttgaaggttggtttctgaatattcattctttagatttgaatattttg

gcattttttgggtgttactgtatttgaagtatgtaaagagtaagaaaaggccaagcatgcatattcccattttat

tgatatgtatgaacaattactgtggatctgaactatcaggtatcagtaactctgtccactaaggttagaacacac

.1450 .1452

gagaagaaataacctagtgttttgt[C/T]t[C/T]tgttggtatttgaacctgagaccttatcatggtcaaccc

.1516

acttgattgacagtaggccacatc[A/C]ttgtgtatatttgtattaattgatggccctcttagttcaaactagg

.1583 .1604 .1606

gatgtcaatatctcaatgaa[T/G]ttagcaattccagtttttca[T/C]g[C/T]ttcgataaagataagagta

.1653 .1663.1664

attaatggtttgtattttctcctgatg[T/A]acattttga[T/C][G/T]gcagatgccataccatcaaaggaa

gcaatgctcttgcaaatatcagttgctatgaaagctatgccgtggtactgtgcccttccatcactttctgagtac

atgattgaaaacggatggaccaaatgttttgcgagaataagtgatgttggatggcctacctacatcatcaatgcg

gctatttatctcgtaatagtggagtttggaatctactggatgcacaagttgttgcatgacataaaacctctgtac

aaatatctgcatgctacacatcatatttacaacaagcaaaacacactttccccgtttgctggtaagctcttaagt

ctagtcataccatattggtgcttatttccgaaaacaacatctagctcgtggagccattaggtttctctgagatac

tctattgattgtttgttgaattaatgagcgtttcctgtgtgatgactaaaaacgttgaaaacctagtcatgagga

cattaaattaatatgtacattttaacatccatgtctttagtcatgcacccttttgtttcgtggtcaggagtgtcg

acttaggtatttggttagactagggaataactgaacacattggaattttgtctcttgccttttaattgtttctgt

atgccaaattcagtttacttgaaaagagtggagtagcgtttgataacttttttgttcttattttctcctttcatt

ttgttggaaactagaacttgtgataggattgaggaaagaaaaatattaagcaaatttctctgaatgtggaaattc

ggaaaaattgctacaaggcatttcctataattccatcacaaagagaaaaagactgtatgttacaactaatgacat

tgtcccttcgattcacacccacaacatgcattcatgtttattaaatcgactctctctgcataccagcccctaacc

aacgcacagcagcataagccctccctatcttctcctccctaccttttgtaacttgtaaccatgctatcatctctg

acattcagaactttctttataactctttgcaatgcatatcctgttctaggaaattggttgctaaatcatcacaat

attgcaagctagctaattcatgatgtccagttaatttatggtttggtatgtgtcacctatgtgttgtaattttct

gacactctatctagctaatttgacctctgagagtgagagtcgtagcttcttttctggtacattttcaagctaatt

.2894

gctaa[A/T]catgttgtgctattcaggattggcattccacccattggatggaatactgcaggcggtgccacacg

.2961 .2967 .3006 .3015

t[T/G]gtagc[T/C]ctattcttggtgccaatgcatttcactacacacatagc[G/A]ctcatatt[C/T]

.3016 .3033 .3060

[G/C]tggaagccttatggac[G/A]gctaatattcatgactgcatacatgg[T/G]aaggtgtggcctgtaatg

.3087(163)

ggtgccgg[C/T]tatcataccattcaccatacaacataccgccataattatggtcattacacaatatggatgga

.3168

ctggatgtttggaactct[T/A]cgtgatcccgttgaagaggatgccaagaaaatgtaatgtgcatgatttgca[

.3220(pyro)

A/C]tcaaggatgttccttgttgttcattgtcctagtaatttgtctcgtgtttccatcgttggtgg

Receptor-like protein kinase (RLPK), PGSC0003DMG400015157

Chr03:51533611..51534733

cgattcacatttcgtaccatgctttataatatagcagaggaaacttctgcaaagaactttcatacagctaaatat

aaacttctcattttggagaggaaacaaaacacacaaggacagtctctcatattggagatgtaacagaatacacaa

.3783(pyro2) .3785(pyro1)

ggaccgtcatacacaagaaata[T/C]t[C/A]tcttagtttcatgacaaacttttatttactgtaagttatcca

ctacatacttaatgcttgatctgcatcaacatgcacctaacatccttactattaggcctctccttaggattctcc

.3914 .3959

agggtacaaaa[A/G]caggcaatcttgagaaccaaaagcatttggtcctcatttccatt[A/G]cccatcagct

.4003

ttggatcaattgctctgtttggatcctcagaag[T/C]catgacatttctcatccatttcactaaactcatctca

.4084 .4106

gaagtgttctggaagaactcatcagatggaagctttcccataa[C/G]tagcacgcctagcagcacccc[G/A]a

.4139

agctgtatatatcacacttatcagtgaactt[C/T]agtgtctgatgatattctggagcgatgtatcctatagtt

cctgccacatttgaagttgtaatatgtgtatgagcatctgggacagcctttgcaaggccaaaatccgcaattcga

.4277

gcttccatgtcatcatcaaggag[G/A]acattgcctggctttagatctctgtgaattatacgctgagtatgatt

.4325 .4379

[T/C]atatggagatactcgagtccagcagctattcccattgcaattcggtgacgtgc[A/T/C]gaccaatcta

.4400(290)

gttcccgtgt[C/T]ccctctctgacttgctggagggtatcctgtaagctcccatttttcatgtactcatagacc

aagtaatggcagtctggtcttggcatatgtgccagtaggggaagcaaattccggtgtctgatttgacctacaatt

.4569 .4574

ttgatttctgatttaatctggcgcattttctta[C/G/T]tcat[A/T]gccttgctatcttcctcagtgagttc

tgcagcatcccttggtggttgtatgatcttctttacggctataatctttccgtcacttccaggtaacgcagcttt

ataaacttttccacatccaccttgcccaatgagttctagtgattccaatccatcttcg

Pectin methylesterase (PME), PGSC0003DMG400009178

chr03:61794617..61794208

gtaactaatttcatgcagccgcggtgggggaaaagttcttggcccgggacataaccttccaaaacacagcaggag

.4496 .4485

cctcgaagcatcaagccgtggcacttcgcgtggggtctgatttgtc[C/A]gcattttata[A/G]atgtgacat

.4475 .4412

[A/G]ttagcttatcaggacaccctctatgtccactccaatcgtcaattctttgtacaatgtttagt[T/C]gct

.4406 .4388

gg[T/G]accgttgattttatttt[T/C]ggaaatggtgctgctgttttacaagactgtgatattcacgccaggc

.4331 .4325

gtcctggttc[G/A]ggcca[G/A]aagaacatggttactgcccaaggaagaactgaccccaaccagaacactgg

.4234

tattgttatccagaaatgtagaattggtgcaacgtctgat[T/C]tgagaccggtgcagaagagtttccct

Squalene monooxygenase (SMO), PGSC0003DMG400004923

Chr04:66508644..66510957

actaagtgaaccgcaagccaatgtgggagaacacaataagaaaaggcaaaaaagaaaacaaagaggaggaaaaaa

.8778

ataaaagtacagagaagagacaaatgtatgattgtacaacttatatttactttccagtt[A/T]atagtgtaatc

aatcatgggaacaaactaaaaaggaaaaagattacaagggatcactagcaaccaaaaattatgcaacagttggac

.8881 .8905 .8913

taaactcttgaaactc[A/T]caaccagtatactgatggaggtg[G/A]tacaatg[G/A]catagccttattct

gtttatagcagttacaacctcacttcaggagggcttcctgtaatatgcaggaacagttgcggggaagaacatctg

cctgaccccttctgccttgatgatagggaaaatgattgcagatgcactctgcatttgaatcaacatgaaattgtc

aaaatacaaggtcacatgggaacacgtgtgtaagattgcaaatattgagcaaaagcaagattgacacctaccgat

attagccgggctccaatccatatacgtttgggtgatgggaaaggaagcagcaagcggccaacaccaaagatagcc

acagcaaagaaatgacaaacgagacttaatggacgagggtttaagccagaaagcaaagatactggtccatctgag

aatactccaccgaggctcaaataatcaaagcatgcttcacgcatctccttcctagcttgatcaggtgaagcacaa

aacaccttatacaaggcaccagccaacgtattgatggtggaggccacaggctgcaggtaaaatatgcgagcagaa

caagtcaatatctataaaagtaattatttttttatttttttttgataaacaatctataaaggtaataatattcta

gttaagtctcaaagtgctgcaggagaattgagtcatcagtcatcctcttttgctctcttttttatttatttattt

atgtaaaagacaagttataggagaattgagtgagtcaagtcctaaaacattgtaatgaaccaagcctttttacat

tatatcgtttttggtagattgatagctggtgaaatcatcaaaggaatgtctgttttgtggttccagatccttcat

tactcactagtcaacatagagtttttacttcactgaggattcttaaagtagaaaacgtggtacttacagcaatca

aatatgttacttatgcaaggaaggatttcaagcgatttttaccttacgcaaggtgtaaaatgactccagatatct

gcaaagagtagatgcatcattcaaatcacgaagaggtttgagaagatcacgcaatacaacaatatcagataatgc

tacagtcattcctccaccagtcaaggggtggcgcatattgaatgcatctcccatgagaagagcaccaggagtagg

.0061 .0067 .0113

atgaggag[C/A]agctggcatgcttc[G/T]gtttggcattgtcctgatgtttcctttatcaattgc[G/A]g

.0115 .0161 .0176

[C/T]aatgaatgcatcttttatctcaggagggacctggaacaaccacac[G/A]aacatctcattgaa[C/T]

.0177 .0188 .0217

[A/G]tcatgcaact[T/A]aagtatgtatactactataatgacatgg[T/C]atgtcattcaagataacgtttt

caacaattctttacctggggagcaactacacttttcaaatatttggccatttcgccattcgaaatagaaggcact

ttttgaccaggtacatcaaccagacagcggacctcagtactgcttatgggatagaacaagataggtgatggatca

gccaaaatgacatgtccatgattttcatgtggaagctggcagttctccaggaccagaccaacaaaacaagaaggg

acttctacctgtacaatattacatgtcattcagagtgttgagagaaacataaggaggggacaacaatgatgatgt

tcatataaaaatatttgttcattcagacaacttcggtacactaattcttattgtctcatacagtagtaagtaaaa

gcaaaagatgatatacaataagtaaaagcaaacgatttcacagatgagctttttaaacaaaagatccttctcaac

.0723 .0725 .0748

aaacaattaatcacccagttccatccttcttat[C/T]t[T/G]aacaaaaatcaagagaaatatt[A/G]ataa

.0759 .0772 .0783

gcgccc[T/A]tacctttgggtc[G/A]caaagggtac[G/A]tcgtagattcgagaaacaaccatcacatacta

.0832

cagttaaaggtgcata[T/C]gctttcaactcttcaccagatttagtcttgtactgaacacctctaatgatcccg

ttttcttcaagcagagacgtaacagtgccttgctctagtttgacactgttaaaaaatcaaagccgatgtgc

Clathrin coat assembly protein AP17 (CCAP), PGSC0003DMG400009862

Chr04:70789222..70790107

ttctctggcacctcagttctgaccccttggggcttttgctagtttttacaaattaaagaatcaaagagatttctc

caacttttttcaattgtttctccagctattttgcctgcagatcttctgaacaaaatggtaactttttcctcttcc

ctttgatctcactgaaaattatttggtagcagaaattacaatctttttgttttccatttcatgaatttgctgttt

tcatttctcttctactgtattgatatttgatcgtttgttttcaactgggtcatttcgaaaatgggtagatccgtt

ttatattgcttcagaacaggcaaggaaagactcgtttggctaagtattatatacctcttgaggaatccgagaagc

acaaggtggaatatgaggtattttagttttttttttgcttttttcttagtttgattatgtgaatttggtgatttt

.9731

agattatgggttttgattatatggtcaatttggatcatgtaggttcatcggttggtggt[G/C]aatagagatcc

.9758 .9776 .9779 .9786

caaattcaccaactt[T/C]gttgaggtaatgaagtc[T/A]ga[A/T]tttttt[A/T]atttcagttgatgga

.9855

agctagcatttttctgattaagcccttttatatctgtggaattgagtttaact[A/T]gatcagcttgaaatgca

.9879

aaaggc[A/G]aaaatgtgattatctgttttgttttcgatctatagaacaccatactaagcgaatttgctgcaat

.9985

tgtgctaaggcaaaggggagactctttcgctagtctttaat[T/G]ttctaaaagtattaatgtttaactatctt

atcgtatttatcttccattgcatcataaatacttgttatcaaaggaataactttgtgaaatgattgaaggatagg

gagtggtgggggattcaa

Biotin carboxylase carrier protein (BCCP), PGSC0003DMG401023454

chr05:51771008..51774510

tgggacgatctgttgccttcaaggttgctcttggtggatcttcaaataattcagctactcctatcacccagtcag

.1121.1122 .1124 .1142

aagaggtgaaatcaccaagtgagacttcatctgcaact[T/C][C/T]g[G/A]tatcagaggaagcaata[T/G

.1190.1191

]ctaactttattagccaagtttcaagccttgtcaagtatgcattccca[T/A][T/G]tttttttcacatctctg

aagagacttttcatgaataatcatgacagactgtcctttgtgcaggctagttgattctagggatattgtggagct

.1335

gcagttaaaacaacttgattgtgaaattctaatccgtaagaaggaggcact[A/G]ccacaaccacctgctcctg

.1381 .1399

ctcctgcacaagctccatttatacaa[T/C]catatcatgtaccatct[G/A]tacaatctaatgcacctcctcc

acctgcacctgcacctgctccaattcaaacccctgcaccttctccagctgcagcaaagtcagctgattcatctct

tccaccgctcaaatccccaatggcagggacattctatagaagtccagcacccagtgaaccagcatttgtgaaggt

attgcatgtccatcttttatgtagtaaattcaaatatggaaacctcaaagattaaaaaaattgaagtttatggtt

taatgcgctctctcaatgcatttggtttccatgatatgcattacaacaggtaggagacaaagtgcagaagggtca

agttctttgcattattgaagcgatgaagttgatgaacgaaatagaggtaaccttttcctttggctattgccattg

cctgctatattgttttcttgtacttatacgagacctctatttcatgacttttaactatttttaaatttatatatc

tgcttttaaattggtgccaaaaaaattgtgtttttacctgcacaacttttagaaaggcattgaaccaaattgaaa

tcgtgattagtaattgtttgctcattcatcaaagaaataatttattgcttctctgctcttctttcccagtgtgga

acagaagtagcatcaatgcatcataactctcaaaagatgcaggaagaaagattgcatttgacttctaatgtataa

acctgctttctacttatacaaacaagcaatgaggtctccaaaaggatgacctagtggttgaggcatgagagatcc

caggttctcattctagctaccacatttgtgtgaccccatgctctagccttgtaataggattacctcgggaaccta

tacctgtgggctggccaggctgcatgatgtattagtgggggtgtgcacaagctagcttgagacaaaaagctttgg

gttgtcattttaccatctaacgactcctttccaaaactgatgtctgttggttttgctagagcttgcaaatttgta

aaagaaaataaacaggagaaatattaagtatctttaaatatatatgtttctactctactagtccagaccacatcc

aatatatatattggacaacctatatatttctagtctaccaaacaaattaagaattttttttcttagactatttac

ttatattattagaagtagtaaaggaagataccccaagattctgggggtttcaatgtatgatgaactggcttagat

tttatattcttttagtggcacttctgggcttgtggattgtttgggcatgctgcatgcttaaactttgatgatact

tagtggatctagtttggacgtgtaaggtttgctatgaaatgtacataatgtttgggaatacctttttctacttga

gattcgtctggtaattggtattgtaacatttttactgtcattgtagacttagcaacatcctttggtgctattcta

atttagaacaacaacatactcggtgtgatcccacaagtgggaggagggcggggtgtactctttttttgttcatta

tttattttggtactgcattattctcttcatttttatgtaataaatatctgattctgtattagcaattcaatttag

aattggcaattggcatagaattgacaatgaaagaaggatgaatgccaattggcatctgttagagggattagtaga

tcagtgaagtcttcttttcatgaagatgagtataagtatatgtcgttgtggttaggaagtgaacaaaatttcttg

caatagctactcaattaattcaggacgccaatattaaagaagttgaggggacttgttccttggaatggttgaagc

tgacagatttagaaataagggaaaaatctaaatcttgttagagaaccataacttctttatttgtattttttgaaa

gaaaaatgtatatgtggagcaggcattcagtgtttattattatataactatactgggaatccataagtttattta

ttttacagcccctcattcttcgttttctaacatgctggttttgtctctgctggagttgtcatgtagatgaactca

tgccagtttcaataaaatgcttgtaagtaagtggcagaagtttcatgtttccttgccctcatgttgcagagaaat

cctttcttgtcgacctgatgaaattttgtggaaactagtggtagttatgactgatgtcgctatttcacctatgtc

gtcccactatgaactaatagttacatcaaagcttgttctactgtatgtgatacttggaagacattagagagttga

tgagagaatgatgataggtttgggctaattttgttacagcattcatttgttgcttcactcaaagacaacactatg

tccattaactccctgattttgcctcctactttagcattatcctcttgatgcgcttttctagttccacatgattgt

ttgatttcagtttcttttagtttgttattgttaagaaaataggtctgggcctaattaaatcccaaaagctagctt

aagaggtgaatgttgcacaacatcatataaggagacaataatctcattccttcaatagacgtaggaggatctaac

atcctccccccgcatgctctagcttggttaactggagcatgaataacgtaaagctgggttctgatatccatgaaa

tggaccttgcccctaacttaacctcaaaatttaggtcatgcaatgaggataaggccacataaggacacaacctat

tccaacctgccaatgtggggcaattgaaaagttatccttgtctaaatttgactgctatgttctatctgttccctt

.4220(Pyro)

caggctgatcggtcaggaaccat[T/C]gttgaggttgttgctgaagatggaaaacctgtcagtgttgatactgt

gagtttcttgactaacctaatccaactattcagtttgttaataccatttccttcctaatatcgtatttatctctc

tgcagcctctgtttgtcatcaaaccatagaaccgttcgggtaagacccaggaaacaatatgcagaggaacttggc

aggtgtttgtttaccttgggcttatgatattttgcagtatttcagtttcagtgagtatgtttgcaacgactggta

gtgtgatggaatagacga

Asparagine synthetase (AspS), PGSC0003DMG400004170

chr06:3216658..3213123

ccaaagtccagaaggacactctatatagtgtcaatagagttgagggtaaaaggaaaaacagtacaattatccaca

.6511(pyro)

aacagcaactagatatcaacttagagcccccacaacttggaacattagtggtgctcaagaacttcaaaacct[C/

A]tatatggttttgatcttttattctagttaaaacattgacccaatgctcttttatgcagcaggatatctaaaag

.6431 .6416 .6413 .6410 .6404 .6399

acgtgc[T/C]gatccttcctataa[T/C]aa[C/T]aa[C/T]aacaa[C/T]aata[A/C]tactgctaatgt

.6316

ttcaaacatcaagtatggacagcaagagtagtgctagttagctccttattgtgagctctgcagccacgcc[C/A]

.6303 .6260 .6253

acgcctaccatc[T/C]ttggcacattattgatgatcggggtgtccaaattcccattag[C/A]aacact[A/G]

gatagatgattgtcataagcagagttatgtacaccgatagcagccctaccggaaggatcaaggttgttcgaccaa

gaagcatcccactcaattgcttttgccgtgctgcaagctatactcggtcctccaggaacggtcaggcttgctgag

ttctgcaaattcaacaaccgatatggtattagtgtttcgagacaccttactacatttgggaactctacaacttta

agcttttcattttaccctggatgaaatattttcgtatatggtactagtgtttcgattttaagagctcgcctgctt

aatattacctgtgggaagaacctctcgaaaatcattctgtaatagtatccttcctttgtagtcggagtgttatgt

gggaagatatgagcagcattaagcatcatcctatcagtcacctaagagacacaaatcgtatacaagatcagctga

cagagtgaaaaaaacaacgaaaactgaacgataatgcttggaatagttttggatttgtgtagtaagctcacatgt

tgttcagcatgtgctttgaggccatcgatccaactatagcctacgccatcgctgaattgttctttctgtctgtac

agaatatgctgcaaaggcacaaaaaacaggacagacatgttagttttaatccataactactggcaaatcagcacg

aaaaatatgacttccaacctttggaaggtacggttgctcctcatcatcaaacgccttcctaagaacccacttctc

aatccttccttgatcatgcttaatctaaaatggacaaaacaagttacagtcaaacccaagtacaacactgttccg

gtgtgatatcttttggttgctatagcaaaatgttgttatagagaacatataatataacttaacatgaataatctg

atacacaaaagaacatggttgttttagtgaaatgttggtatagaggatgattgttataaaaaggtctgactgtac

agagaaagcatatcgagttatacgtctattctcatcaatgattcgagcttagtttcttaccatcttccattcggg

atcgatactcatggcaacatcgatgaactctttatccagaaatggtactctagcttctaagccccacgcggatgt

.5091 .5079

agccttgtttgctcttaaacagtcatactggtgaag[C/T]gcttttatctg[C/A]gcgtataaatgaagaaca

.5051 .5026.5025

agatcagca[T/G]tttttaaaactcgtcctgtctcaa[T/C][G/T]aggttcgaaacaagaaggcaatattcc

.4992

aagaa[C/T]gattattaccttgcgacatgtttccgtgtggaactcttccttgttgggagccttgtggaagtaca

agtaaccaccaaaaatttcgtcagcgccttcccctgatatgaccatcttcactcctagtgatttaatcttacgcg

acataaggaacataggagtgctggctcttattgttgttacatcatacgtctcgatatgatatataacatcttcaa

tagcatcaataccgtcctgcaaatatgaaacgcgttgtttgacaacatgaaaacagaaggtaatcccgagatcaa

ataaaaagtaacaccgcgatttacaaacctgaacagtaaagtgaaactcatggtgaacggttcctaaaaagtcag

caacttcttttgcagccttgagatctggtgagccctgttcaatcgtaaaacacaaaaatttataaatgcacaact

gaaaaatgcaacgggcacgtataggttgaaaccatcaagtttaaattcttgatccgcttctggagtgactcacct

cgagaccaacacagaaggaatgaagttgtgctccccattgcttagcagcttttgttccagccaagtatcgagtag

tgacagaagcaaccaaagacgaatcaagtccccccgagagcagaacgccaaaggggacatcagtcatcaaccgtt

tgataacagcctacaaaataacaaccactcattaagatcaacagacttctcgggtactaagacccaaagtcaaaa

ggaaaacagcctagattaattcatcccaaacaaaactaataccaaataataacaaatactcacattttcgaaggc

acgcctcagaaccaaagtgtcataaggagtggaaggaattgcttcagagaaccaagcgggattgtaccatctcct

aagccctccgttcttgctagagtacaagtgccccggagggaaaacttcaaaatgttcacaatcatcattcaagcc

cttcagctcagatgatatccacacagagcctgtgacacacaataacatatatcacttctagtttgttatgttgtt

cagaccctccaaaaatgaactcgtgtcacatttttgaagagtacgagcaacataattagtttctatttggaaaat

ttgtataaaaaatcctaccatcaagtccccaaccaatatagaggggtgtaattccgatggcatcacgagcagcaa

gaaagctattatcgcgagtatccaataatacaaaagagaacactccatccagcatgtcaacaaaattttctccat

attcttcatactgatcaacacatatattaaaactcaagtaaaccaaaaattacttataagttaatttaaccagct

tataagcgtatctaaacgccccgatgtttctattataattcactagcctaagttggttgataaataaagtaagac

tatatactcacaagatgagcaataacatcacaatcacttccagtcctaaacttgtgattaggcataagttttcga

agtttttcatgattgtagatctctccattaacctgcaaaattcgacacaaaattaatcatcaaacaaatttttca

tccttatgtaagtctaaaattatgttgcttccaaactcttcaaaaatatcgactagtgcctgccagatcctccaa

.3305 .3285

aagtagcacattttcgaaagacatgacacatacatacacgacaagt[C/T]gacaacatttttgaagagt[C/T]

.3283 .3277 .3242

c[G/A]aacaa[C/T]agtagagtagaggtgtactcacagtaacaacaat[C/T]tttttgtcttcattaaacag

.3215 .3197

aggttg[A/G]tcaccagaagcagggtc[G/T]ataattgctagacgttgatgtgccaagtaaaaatcaccatat

tgaaatattccactccaatccggtccacgatg

Chloroplast protease (Chp), PGSC0003DMG400017311

chr07:50424348..50424970

.4380

gctccactgaaaccaggtgtcctcatggctat[G/A]acttcaagagacacatctgaatcaaacttcttgttgcc

.4478.4479

ggcatgaacctttaagatctctgttcttcccttgatgtctggaacatccacggatacct[A/G][G/C]ataaga

.4512 .4515

gctgtaaattttagtttcttgcaaaa[G/T]tc[T/C]cagttaaaaaacggaggcaaggacagatgcttacttg

tctatcaaatcgtcctggcctcagcaaagcagagtcaagaatatctgcacgattggttgccgcaacaactattat

accagtatttccttcgaaaccatccatttcagtcaatagttggttcagggtctgttccctttcatcatttcc

.4700

[T/C]cctccaattccagtccctctttgccgcccaacagcatcaatttcatcaacaaatacaatgcaaggagcat

tttccttggccttcttgaaaagatcacggactcgagaggctccaacaccaacaaacatctcaacgaattctga

.4844 .4871

[G/A]cctgaaattgagaaaaatggaacacc[T/C]gcttcaccagcaattgcctttgctagcaaggttttccca

gtaccaggaggaccaacaagaagaacaccttttggaatacgagcccccactgcagtaaat

Avr9/Cf-9 rapidly elicited protein 20 (ACRE20), PGSC0003DMG400025023

Chr10:1636342..1637559

gaaccacgggccgacatatttggtctgtcttctggaatctgctggacacataacagacttacatgtatgcaccgc

gatatttccaacatagagcttgtgtcctctaacgaggaatcaactaacgcgaggggtgtcccttccgtccacatt

ctccaagcctgaaaaggggaataaagaaagaaaggcacattacataaccaaacattcaaacttggcttcagatga

caatttagcactccaattttgactatgcacatctagacagttcaactcgtctccactgtgtcagttgaacactcc

aacttcgtctttactgtaaccttgttctttacttccacggatagtgactagtgagatattaactcattgactcta

aactcattccaattatacctatattccacacgctgagaaataaatgtcattgacttgcaatgcttgtgatttctc

.6830 .6835

ataagaagaagcatcaaaactcaaaagaaagagaaaga[T/G]tttg[C/T]gtacaacaactacgcctcaatcc

caaacaagtggtcagctatatgaatccccagttcttcattaaagctcattttcatatcctcatcgtgtgcattaa

.6975

cgaaaaaatctaataaaagtttcagcttagaatttatcaaa[C/G]agtgagtttcagacattgaatggtta

.7002

[A/G]atcataaggaaaaaaaagtgaaaagacccttggtagagacaatagagagaactgatgaaataagctgttt

.7092

ctccatagatacaactttc[C/T]atatatccctatccatcaataacggaaaccctttacacagctcgtctatca

.7156 .7181 .7206

aaccaaccccac[G/C]taatttctcatacaaaataggtaa[C/T]aaatactcatccgtagagacagtt[A/G]

agataactgattaaacctattgtttctccattagtacaactttctgtatatgtttttgcaatgtattacatgttt

.7289.7290

acataga[C/T][A/G]aaagtataaaacgacaaaagttacaacgaaaagttgaattcttggtaatgagattctt

tctaacaaaaactgagactctcaaatgtgatgaccccttcatccctatccatcaacaactgaaaccctttacaca

gctcgtcaatcgaaccatccccacttagtttttctcatccatattaggcaacaaatagttgaacatgattacaat

aattcagtgtataactataagaattttatcataaaatgtgcggttctgctcttatctcgat

Magnesium-protoporphyrin monomethyl ester [oxidative] cyclase, chloroplast (MPP), PGSC0003DMG400007188

Chr10:59602799..59602264

ggaagatgttttgtcaagtgcatgttggccctctgagtcttgactcgtcaaagaagaaaaccttcatcaattgaa

.2687.2686 .2656

gaatgcctaataaatgagaccagt[G/A][A/T]agatgaaaacttttaatgatgtactcacaa[G/T]aaattc

.2634 .2609 .2601(pyro)

attctagataatacaagggagtgtcct[C/T)tatgcaggaaactgtttcttgaat[A/C]cactata[T/C]aa

.2580 .2575 .2556 .2537

cttacaacaatgaacatg[G/C]acct[C/T]tatgtgaaaacaaaacaa[T/C]cgagattctgtttctcgt[G

/A]aaaaggaaattagtaggtaagttgtggttcaaactctgcaaaatcaactgaacctgactcaataggtttcat

.2458

gagata[C/T]gcagccaataactcagaagccaaagctgcaatcagagggatctttttgaaattcttcaccagtg

gaatctcatcactctcactaacagcaattagtttggtgttaatctccaccatcctgtccaacttcctcttgaatt

ctgggttctcaacatcaaggacagcagggaaaatccttgcagttgtgcggtttgt
